# Arabinoxylan and pectin features differentiate maize inbred lines with contrasting Fusarium stalk rot resistance

**DOI:** 10.3389/fpls.2026.1840041

**Published:** 2026-06-30

**Authors:** Alba Manga-Robles, Diego Rebaque, Pilar Carrancio-Jato, Antonio Encina, Rogelio Santiago, Francisco Vilaplana, Penélope García-Angulo

**Affiliations:** 1 Área de Fisiología Vegetal, Departamento de Ingeniería y Ciencias Agrarias, Universidad de León, León, Spain; 2 Institute of Molecular Biology, Genomics and Proteomics (INBIOMIC), Universidad de León, León, Spain; 3 KTH FOOD, KTH Royal Institute of Technology, Stockholm, Sweden; 4 Universidad Politécnica de Madrid, Madrid, Spain; 5 Misión Biológica de Galicia, Sede de Pontevedra (CSIC), Pontevedra, Spain; 6 Division of Glycoscience, Department of Chemistry, KTH Royal Institute of Technology, AlbaNova University Centre, Stockholm, Sweden

**Keywords:** cell wall, *Fusarium graminearum*, polysaccharides, subcritical water extraction, linkage analysis, stalk rot, *Zea mays*

## Abstract

Maize is susceptible to stalk rot caused by *Fusarium graminearum*, which reduces the forage quality and yield. This fungus spreads within the host, degrading pith cell walls and colonizing all other plant tissues. The aim of this research was to investigate genotypic differences in cell wall composition of two inbred lines (B73 and EA2024) with different resistance to Fusarium (susceptible and resistant respectively). We also compared the cell wall composition and structure of pith tissues between Fusarium-inoculated and non-inoculated plants. Cell wall analysis of non-inoculated plants showed no quantitative differences in major components between B73 and EA2024. However, quantitative variations between inbreds were detected in minor cell wall components including *p*-coumaric acid, ferulic acid, diferulic acids and mixed-linked β-glucans. Detailed structural analyses by subcritical water extraction of loosely-bound native polysaccharides indicated that EA2024 presented a pectin-enriched population, more mannans and callose, whereas the Fusarium-susceptible inbred B73 showed higher tightly-bound disubstituted xyloses in the arabinoxylan population. The infection scarcely affected the native composition of the cell wall in either of the two lines, while it did affect the degree of arabinoxylan substitution. The results obtained may contribute to a better understanding of the spread progression of Fusarium and the identification of cell wall markers to be used in the screening for new stalk-rot-resistant genotypes.

## 1 Introduction

Maize (*Zea mays* L.) is an extensively cultivated crop worldwide and offers a wide range of products utilized for a multitude of purposes (Zhang et al., 2022). Although it is a very productive cereal, several factors such as fungal diseases reduce grain yield and quality every year (Juroszek and Von Tiedemann, 2013). One of the most relevant fungal diseases of maize is stalk rot produced by *Fusarium graminearum* (Jackson-Ziems et al., 2014; Santiago et al., 2007). Additionally, *F. graminearum* generates toxins such as deoxynivalenol and zearalenone (Jedidi et al., 2021; Mesterházy et al., 2012; Quesada-Ocampo et al., 2016; Sumarah, 2022), harmful to both humans and animals (Pestka, 2010; Trail, 2009; Wang et al., 2020). This fungus exhibits the greatest pathogenicity and aggressiveness compared to other species that produce stalk rot (Broders et al., 2007; Gai et al., 2017; Santiago et al., 2007). *F. graminearum* naturally infects maize plants through roots or wounds in the stalk caused by corn borers, hailstorms or mechanical damaging (Zhang et al., 2016). Upon infection, *F. graminearum* sequentially releases cell wall degrading enzymes (CWDEs) with two main purposes: obtaining nutrients from cell wall polymers and facilitating their spread along pith tissue (An et al., 2005). *F. graminearum* has a hemibiotrophic lifestyle (Brown et al., 2017): once inside the internode, it begins to develop along the pith, firstly growing between plant cells, and then both inter- and intra-cellularly, and over time, this tissue degrades keeping the vascular bundles undisturbed (Zhang et al., 2016; Zuo et al., 2022). However, the final stages of infection may lead to lodging (Hansey et al., 2010). The means by which this fungus surpasses numerous physical obstacles and the active defenses of the host, gradually establishing itself within the stalk, remain poorly understood (Zhang et al., 2016).

The inbred lines used in this study were selected based on previous research conducted by our group, which demonstrated that lodging resistance is positively correlated with stalk strength. According to our predictive model, H-type lignin subunits contribute to greater rind penetration strength, while the content of ferulic and diferulic acids influences susceptibility to lodging (Manga-Robles et al., 2021). Based on these findings, B73 is classified as an inbred line with high mechanical stalk strength and strong lodging resistance, whereas EA2024 exhibits low mechanical stalk strength and is more susceptible to lodging. Interestingly, these two inbred lines display contrasting responses to drought stress, with EA2024 being more resistant than B73 (Calderone et al., 2024).

The mechanisms underlying disease resistance are multifaceted in cereals and several components may play a role (Rampersad, 2020; Santiago et al., 2007), which makes it difficult to obtain resistant genotypes. This is the reason why most maize inbreds and hybrids are susceptible to *Fusarium* spp (Kikot et al., 2009; Mesterházy et al., 2012; Quesada-Ocampo et al., 2016). However, several studies indicated that plants with modified cell wall composition showed altered disease resistance against pathogens (Houston et al., 2016; Molina et al., 2021).

The plant cell wall is the outermost semirigid layer of plant cells, enclosing the protoplasm, and provides essential structural and physiological functions such as cell shape differentiation, tissue adhesion, cell-cell recognition and, of particular interest for this research, the ability to respond against biotic and abiotic stresses (Houston et al., 2016; Zhang et al., 2021). Cell walls are macromolecular nanocomposites mainly consisting of cellulose, lignin and matrix polysaccharides (hemicelluloses and pectins) (Kozlova et al., 2020; Zhong et al., 2019). Not only the composition but also the cell wall structure, the location of specific microdomains and the spatiotemporal change on the distribution of some components determine important cell functions (Gkolemis et al., 2023). For instance, the cell wall is a key defense barrier for each plant cell, whose structure and composition can be modified during infection. Besides, the cell wall contains antimicrobial compounds released during its degradation (García-Olmedo et al., 2001; Schulze-Lefert, 2004; Vorwerk et al., 2004). In line with this, the role of cell wall integrity maintenance plays a strategic role in triggering responses to developmental or external stimuli (Engelsdorf and Hamann, 2014; Wolf et al., 2012). The alteration of cell wall integrity due to pathogen invasion or wound damage leads to the release of signaling compounds referred to as Damage-Associated Molecular Patterns (DAMPs; Cantu et al., 2008; Vorwerk et al., 2004) able to induce a pattern triggered immunity (PTI). Besides this, plants can activate a subsequent layer of defensive responses based on the recognition of pathogen-derived effectors, the so-called effector triggered immunity (ETI). Both responses are associated with intracellular signaling pathways that lead, among others, to cell wall remodeling (Zhou and Zhang, 2020).

The relevance of cell wall composition in determining the resistance of plants against a plethora of pathogens with different parasitic styles has been conclusively shown (Molina et al., 2021). Although this topic is a growing field of research, most of the studies have been carried out with the model plant species *Arabidopsis thaliana*. However, few studies address the role of the cell wall in plant-pathogen interactions in maize under field conditions. Previous publications have indicated that the resistance to *Fusarium* spp. appears to be related to the cell wall composition of maize pith, the inner and soft tissue found in maize stalks (Santiago et al., 2007; Cacique et al., 2020; Salcedo et al., 2021). However, this relationship, particularly in those aspects related to the detailed structure of cell wall polysaccharides, deserves further study.

Given its highly complex structure, the in-depth analysis of the cell wall is challenging and requires multiple techniques. Traditionally, classical chemical-based extractions were used to fractionate different pectic and hemicellulose polysaccharide populations by using calcium chelators, de-esterifying agents and alkali. The use of these conventional methods causes extensive depolymerization and the loss of sugar substituents from the polysaccharide backbone (BB) (Rudjito et al., 2020; Martínez-Abad et al., 2020; Rincón et al., 2021). Due to the importance of knowing the structure of the polysaccharides *in muro*, keeping their molecular substitutions, an alternative extraction methodology based on aqueous extractions has emerged. In this framework, the subcritical water extraction (SWE) method has been presented as a technology which preserves the native molecular features of cell wall polysaccharides. This “green” method, widely exploited for plant lignocellulose biomass (e.g. Martínez-Abad et al., 2020), uses pressurized water together with high temperature conditions to isolate cell wall polysaccharides based on their extractability and recalcitrance in the lignocellulosic scaffold.

In this framework, B73 and EA2024 maize inbred lines, previously assayed for other stresses in our group (Manga-Robles et al., 2021; Calderone et al., 2024) were inoculated/non-inoculated with *F. graminearum* in a field experiment. The disease index was evaluated at maturity stage, and the spread of *F. graminearum* in pith tissue indicated significant differences in the damage produced by the fungus between inbreds, being B73 more affected than EA2024. The main objective of the current study was to provide information on the detailed composition and native molecular structure of the cell wall matrix polysaccharides from these two contrasting maize inbreds under control and *F. graminearum* inoculation conditions.

## 2 Materials and methods

### 2.1 Plant material

Maize inbred lines, B73 and EA2024, from the germplasm bank of Misión Biológica de Galicia-CSIC (Pontevedra, Spain) were grown in 2022 on a basic sandy loam soil in Pontevedra, Spain (42° 25´N, 8° 38´W, 20 m above sea level). Two trials were carried out at two sowing times [standard sowing in trial A (May/05/2022) and late sowing in trial B (June/03/2022)], using a split-plot design with three replicates. Main plots correspond to the genotypes and subplots to the treatments (control or inoculated). Each experimental plot comprised two rows (25 seeds in total) with a separation of 0.80 m between them and seeds were positioned at intervals of 0.18 m resulting in a plant density of 70,000 plants/ha. Irrigation, fertilization and weed control were carried out following the regular agronomic practices used in Misión Biológica de Galicia-CSIC (Spain).

### 2.2 Fungal growth conditions and inoculation

*Fusarium graminearum* freeze-dried isolate was acquired at the Westerdijk Fungal Biodiversity Institute (Utrecht, Netherlands) from the CBS Culture Collection 123657 (20.5297), EXB22-0092. The fungus was cultured in Petri dishes with potato dextrose agar medium (PDA). Sub-cultures were obtained and when developed, pieces of 1x1 cm were transferred to Bilay´s modified liquid medium for sporulation: KH_2_PO_4_ (2 g/l), KNO_3_ (2 g/l), KCl (1 g/l), MgSO_4_ (2.05 g/l), Dextrose/Glc (1 g/l), FeSO_4_ (0.4 g/l), FeCl_3_ (0.3 g/l), MnSO_4_ (0.2 g/l) and ZnSO_4_ (0.4 g/l). After 9 days of air bubbling, this suspension was filtered and macrospores were recorded in Neubauer counting chamber. *F. graminearum* inoculum was prepared with sterilized distilled water at a final concentration of 2.5 x 10^5^ macrospores/ml.

In the inoculated plots, plants were inoculated 10 days after silking (50% of the plants of the plots showing silk) with the spore suspension previously described (2 ml/plant). The inoculator syringe was placed at a 45° inclination of the stalk, in the center of the second internode above the crown roots (Santiago at al. 2007). In case of control plants, Decis Evo insecticide (Deltametrin 2.5%; 5 ml/10 l water) was manually applied 3 times every 14 days on all plants to maintain a stable phytosanitary reference and minimize indirect damage caused by insects that could compromise their status as plants unaffected by Fusarium.

### 2.3 Phenotypic damage traits

#### 2.3.1 Damage index and sampling

After 10 and 25 days post-inoculation, stalks from both trials and both conditions, were collected and fungal development through the pith was recorded using a 0 to 8 visual damage scale from Santiago et al. (2007) with minor modifications, in which: 0 = No spread from the wound area; 1 = 25% of the inoculated internode affected (1% - 25%); 2 = 50% of the inoculated internode affected (26% - 50%); 3 = 75% of the inoculated internode affected (51% - 75%); 4 = 100% of the inoculated internode affected (76% - 100%); 5 = Infection scattered to one internode adjacent to the inoculated one (mild); 6 = Infection in both internodes adjacent to the inoculated one (above and below) (mild); 7 = Infection in both internodes adjacent to the inoculated internode (severe); 8 = Severe infection scattered spreading along the stalk internodes (**Supplementary Figure 1**). Scoring was performed by a single trained evaluator using coded samples, blind to genotype identity at the time of assessment.

The second internode below the main ear was collected from 5 plants for each condition and replicate. The rind was manually removed, and the pith was freeze-dried and powdered with a rotor mill (Pulverisette-14, Fritsch).

### 2.4 Fungus detection qPCR

For DNA extraction, pith tissue was collected from one randomly selected plant within each plot. Genomic DNA was extracted using the NZY Plant/Fungi gDNA Isolation Kit according to manufactured instructions. DNA samples were concentrated in a speed-vac for 30 minutes at 40 °C. The presence of fungus in the samples was quantified by qPCR using the PowerUp SYBR Green Master Mix Kit (Thermo Fisher). The UBCQ21 gene was chosen as the housekeeping gene (Manoli et al., 2012), and primers were designed based on the 16N-conserve fragment of *F. graminearum* (NC_026474.1) to detect presence in the samples (**Supplementary Figure 2**).

### 2.5 Cell wall analysis

Pulverized material from pith stalks was used to isolate cell walls following the protocol described by Rebaque et al. (2017).

Cellulose quantification was performed accordingly to the method described by Updegraff (1969) employing the hydrolytic conditions reported by Saeman et al (1963). Released glucose from cellulose were determined by the anthrone method (Dische, 1962), where 0.5 ml of each sample was mixed with 1 ml of 0.2% (w/v) anthrone in 96% H_2_SO_4_. Finally, the absorbance of each sample was measured at 620 nm and the quantification was performed using a standard curve of glucose.

The cellulose crystallinity index (CRI) was determined using a Bruker Discover D8 diffractometer following the procedure outlined by Segal et al. (1959). Pith cell walls were analyzed in reflection mode with Cu K-α X-rays (wavelength of 1.5418 nm) over a scanning range from 2θ = 10-50 ° with a step width of 0.0205 °. The method involved recording the minimum peak intensity at 2θ between 18°-19° (IAM) and the highest peak intensity at 2θ between 22 °-23 ° (I200). The crystallinity index was then calculated using the formula CRI = [1 - IAM/I200] * 100.

Klason lignin content was determined following the protocol reported by Dence (1992) with minor modifications. Briefly, 50 mg of pith cell walls were hydrolyzed with 2 ml of 72% H_2_SO_4_ and incubated at 30 °C for 1 h. Subsequently, H_2_SO_4_ was diluted to 2.48% by adding 56 ml of distilled water and autoclaved at 115 °C for 3 h. After cooling, samples were filtered through glass fiber filters (Whatman GF/A) and the residue was kept at 60 °C for 24 h. Finally, samples were weighed together with the filters and then the weight of the filter was subtracted obtaining the Klason lignin content.

For esterified hydroxycinnamate analyses, the content of ferulic acid (FA), *p*-coumaric acid (*p*CA) and dehydrodiferulate isomers (DFAs) was carried out by HPLC as previously described (Santiago et al., 2018).

Ester-linked acetic acid quantification by HPLC was conducted following Martínez-Abad et al. (2018) procedure.

### 2.6 Monosaccharide composition

Pith cell walls (2 mg) and their corresponding freeze-dried subcritical fractions (0.1 mg) were hydrolyzed with 2 M trifluoroacetic acid (TFA) for 3 h at 121 °C. Then, the hydrolysis products were air dried, resuspended in water and filtered through PTFE filters (45 μm). The released monosaccharides were analyzed in a LC 930 Compact IC Flex and 889 IC Sample Centre (Metrohm) chromatography system equipped with an IC pulsed amperometric detector (FlexiPAD). Chromatographic separation was carried out using a Metrosep Carb 2 250/4.0 analytical column (Metrohm) and a Metrosep Carb 2 Guard/4.0 guard column (Metrohm) at 25 °C. The eluents used for the high-performance anion-exchange chromatography with pulsed amperometric detection (HPAEC-PAD) were a mixture of 1 mM NaOH with 1 mM sodium acetate (eluent A) and a mixture of 100 mM NaOH with 170 mM sodium acetate (eluent B). Monosaccharides were separated at a flow rate of 0.5 ml/min with the following chromatographic method: 100% eluent A (0–24 min), 100% eluent B (24.1–55 min) and 100% eluent A (55.1–75 min). The monosaccharides quantification was carried out using standard curves of rhamnose (Rha), arabinose (Ara), galactose (Gal), glucose (Glc), xylose (Xyl), mannose (Man), galacturonic acid (GalA) and glucuronic acid (GlcA). Arabinoxylan (AX) content was referred to as the sum of Ara and Xyl molar percentages.

Subcritical water extraction (SWE) of the cell walls was carried out by using an Accelerated Solvent Extraction DionexTM ASETM 350 (Thermo Fisher Scientific Inc., USA) following the procedure reported by Rudjito et al. (2019) with modifications. In short, extraction cells were loaded with 3 g of pith cell walls and 1 g of ASE Diatomaceous Earth (Thermo Fisher Scientific), and ASE Extraction A cellulose Filter (Thermo Fisher Scientific) was placed at the bottom of the cell extraction. Since cell walls used as starting material presented acetyl groups (0.1- 0.14 mol acetic acid/mol xyl), acetate buffer 100 mM pH 5 was used to avoid polymer degradation by acetic acid formation upon extraction (Rudjito et al., 2020). Then, samples were sequentially extracted at 160 °C at 10 (10-SWE) and 30 (30-SEW) min. Throughout the extraction process, the extractor maintained a pressure range of 10–11 MPa and a solid to liquid ratio of 1:14 (w/v). The pH stability was checked in all the fractions after extraction. The obtained fractions underwent dialysis using 3.5 KDa membranes (MWCO Spectra/Por; Spectrum Laboratories Inc., Rancho Dominguez, CA, USA) and tap water for 72 h. Then, extracts were freeze-dried and weighted to calculate extraction yields.

Starch quantification was performed using the K-TSTA-50A Total Starch kit (Megazyme, Wicklow, Ireland) and absorbances were measured at 510 nm and standard curves of Glc were used for quantification.

The content of mixed-linked β-glucans (1,3;1,4 β-glucans; MLGs) was measured using the K-BGLU β-Glucan (Mixed Linkage) kit (Megazyme, Wicklow, Ireland) and absorbances were measured at 510 nm and standard curves of Glc were used for quantification.

### 2.7 Glycosidic linkage analysis

Linkage analysis of polysaccharidic samples was carried out by means of per-O-methylation, hydrolysis, derivatization and further analysis using GC-MS, as previously reported (Pettolino et al., 2012). The separation of the compounds was carried out using the method already described by Rudjito et al. (2019). Values regarding partially methylated alditol acetates were corrected to the monosaccharide composition referred as molar percentage. Linkage analysis was carried out without the previous carbodiimide treatment thus acid residues were not detected in the chromatograms. In case of Fuc and Rha, there were no standards.

### 2.8 Statistical analysis

After verifying that the data followed a normal distribution using the Kolmogorov-Smirnov test (*p* < 0.05) and the homogeneity of variances by a Levene test (*p* < 0.05), a T-student test and one-way ANOVA followed by Tukey’s *post hoc* test were performed using SPSS software (p ≤ 0.05; IBM Corp. IBM SPSS Statistics for Windows, v.26).

## 3 Results

### 3.1 Phenotypic traits

**Figure 1:**
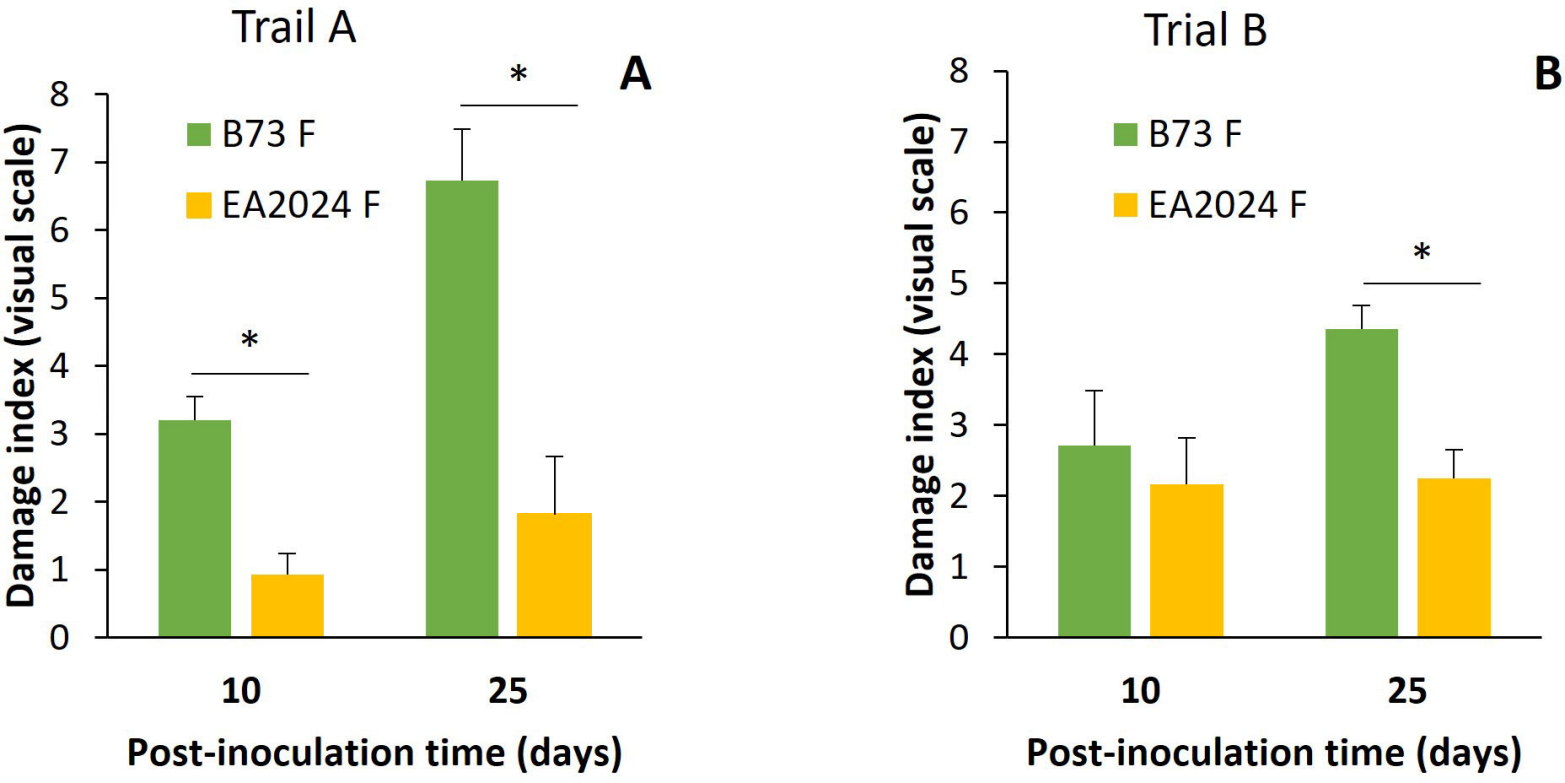
Damage index of the stalks infected with *Fusarium graminearum* in B73 and EA2024 maize inbreds. Maize plants with silking flowers at two different trials **(A, B)** were analyzed at 10 and 25 days post-inoculation with a macrospore solution (2.5 x 10^5^ macrospores/ml) of *F. graminearum*. Visual scale from 0 (no spread from the wound area) to 8 (severe infection scattered along the stalk) is represented. Data represent x ± SD (n=3). Asterisks indicate significant differences (p ≤ 0.05) according to t-Student test.

After 10 and 25 days post-inoculation (dpi) with *F. graminearum* macrospores suspension, the maize stalks from the two trials were split for damage. Fungal development through the pith was recorded using a visual scale from 0 (no spread from the wound area) to 8 (severe infection scattered along the stalk); also, for control conditions (B73 C and EA2024 C). The damage index was significantly higher for B73 compared to EA2024 at 25 dpi in both trials (B73 F and EA2024 F) (**Figure 1**). Regarding 10 dpi, B73 showed significant differences exclusively in trial A. In addition, fungal damage along the pith tissue increased over time after inoculation for B73, whereas it remained almost unchanged in the case of EA2024.

To distinguish *F. graminearum*, the expression of a conserved fragment of 16N sequence was analyzed in the second stem internode below the main ear of B73 and EA2024 under control and infected conditions after 10 and 25 dpi. The fact that the experiment was conducted under field conditions explains why the fragment was detected at low level even in some control samples, but only B73 showed a high presence and symptoms of medullary damage at 25 dpi (**Supplementary Figure 2**).

Early stages of inoculation (10 dpi) showed differences between inbreds just in the standard sowing (the trial A), probably related to the favorable climatic conditions of the period. On the other hand, data from both trials allowed us to classify B73 and EA2024 as susceptible and resistant inbreds to *F. graminearum* at 25 dpi, respectively. As the largest difference between inbreds was found in the standard sowing (trial A) at 25 dpi, these samples were selected for further analysis.

### 3.2 Cell wall composition characterization

During the infection, *F. graminearum* usually spreads along the pith of maize stalks, therefore, the study of this tissue may be relevant to better understand this process. In particular, cell wall composition of this tissue was analyzed to examine its influence on the two maize inbreds, B73 and EA2024, after 25 days post-inoculation.

**Figure 2:**
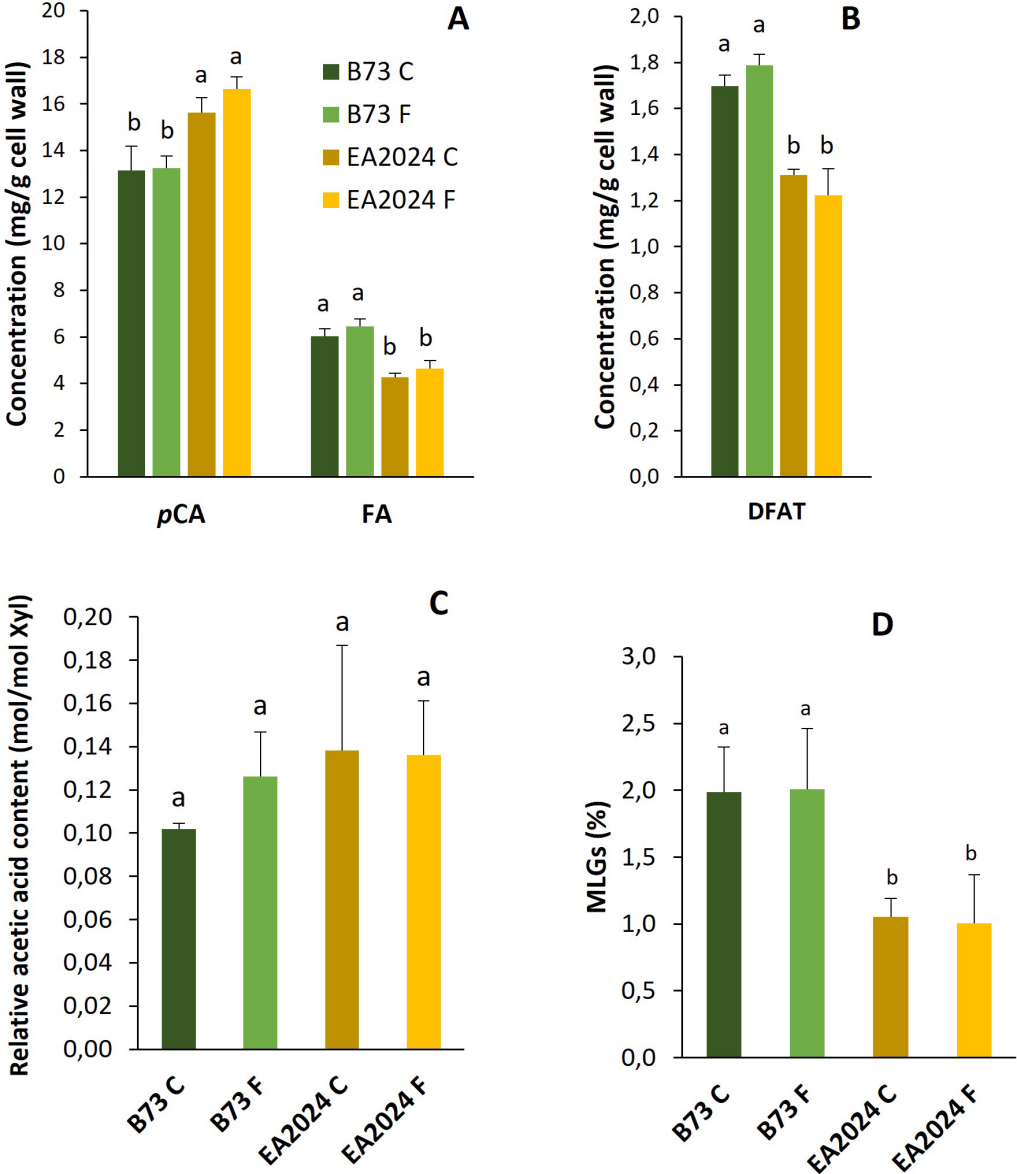
Hydroxycinnamate, degree of acetylation and mix-glucan content of pith cell walls. Analysis was performed in the second stem internode below the main ear 25 days after inoculation with a macrospore solution (2.5 x 10^5^ macrospores/ml) of *F. graminearum* in the center of the second internode above the crown roots. **(A)** *p*-coumaric acid (*p*CA), ferulic acid (FA), **(B)** total diferulates (DFAT) and **(C)** relative acetic acid contents and **(D)** mixed-linked β-glucans (MLGs) percentage of pith cell walls from B73 and EA2024 maize inbreds grown in control -C- and *F. graminearum -*F- inoculation conditions. Data represent x ± SD (n=3). Statistical comparisons were performed independently within, but not between, different compound groups using one-way ANOVA followed by a *post hoc* Tukey test (*p* ≤ 0.05).

In general terms, no changes were observed in the major components of the cell wall following infection with *F. graminearum* in either of the two lines analyzed (**Supplementary Figure 3**). However, EA2024 showed significantly higher levels of esterified *p*CA than B73, whereas FA and DFAT were more abundant in B73 (**Figures 2A, B**). *F. graminearum* infection did not affect cell wall–esterified hydroxycinnamate levels in either inbred (**Figures 2A, B**; **Supplementary Figure 4**). The degree of acetylation was also unchanged by infection (**Figure 2C**). EA2024 contained lower levels of mixed-linked β-glucans than B73 (1% vs. 2%), with no infection-related changes in either line (**Figure 2D**). The highest starch content (2.5%) was observed in infected B73 (**Supplementary Figure 3E**).

**Figure 3:**
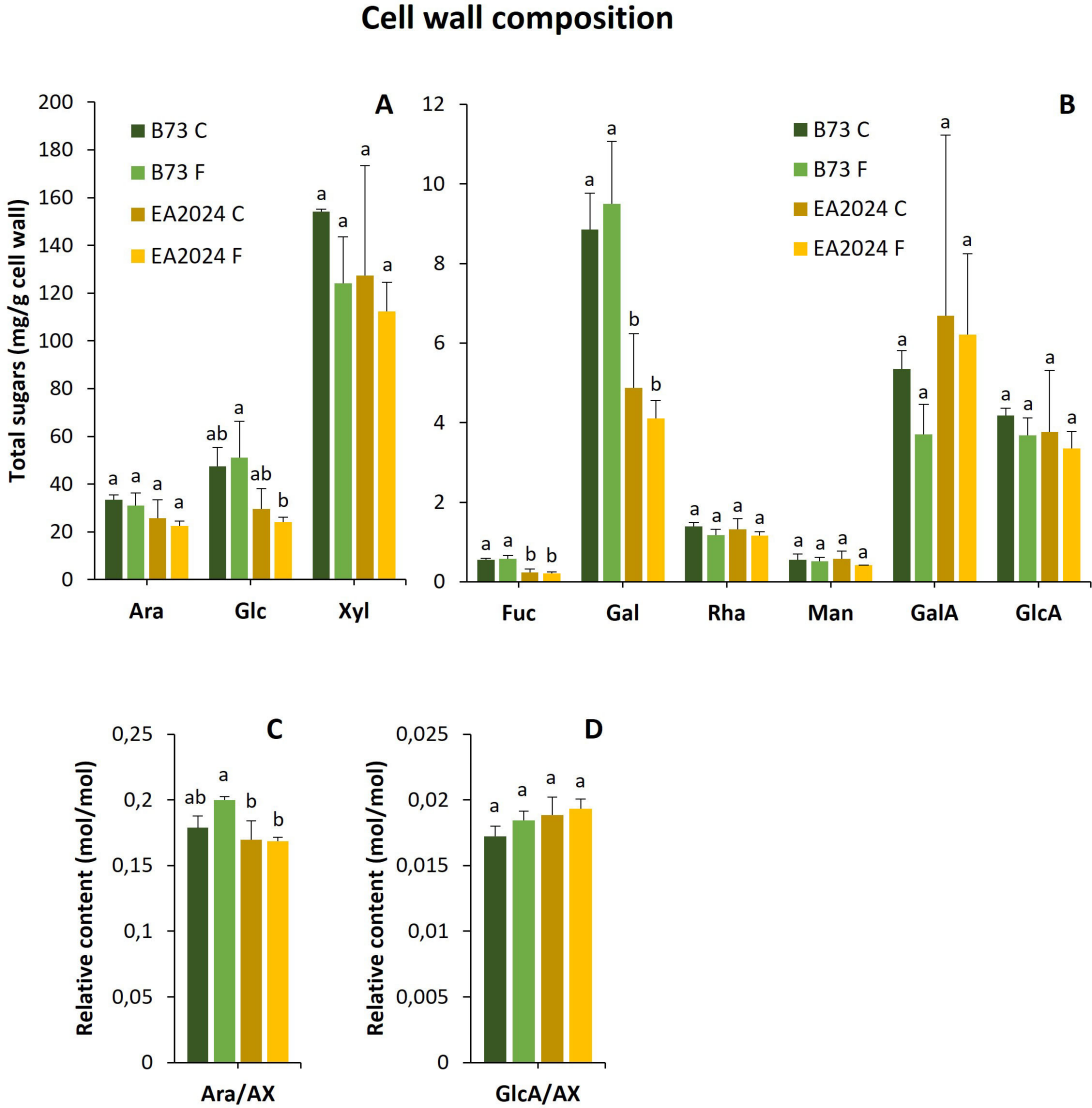
Monosaccharide composition of pith cell walls of B73 and EA2024 maize inbreds after *Fusarium graminearum* infection. Analysis was performed in the second stem internode below the main ear 25 days after inoculation with a macrospore solution (2.5 x 105 macrospores/ml) of *F.graminearum* in the center of the second internode above the crown roots. **(A, B)** Monosaccharide composition and **(C, D)** molar ratios of substitutions of AX (arabinoxylan referred to as Ara + Xyl) of pith cell walls from B73 and EA2024 maize inbreds grown in control -C- and *F. graminearum* -F- inoculation conditions. Monosaccharides are referred to as: Ara (arabinose), Xyl (xylose), Glc (glucose), Gal (galactose), Rha (rhamnose), Man (mannose), GlcA (glucuronic acid), GalA (galacturonic acid). Data represent x ± SD (n=3). Statistical comparisons were performed independently within, but not between, different compound groups using one-way ANOVA followed by a *post hoc* Tukey test (*p* ≤ 0.05).

Xyl was the predominant monosaccharide in pith cell walls, followed by Ara and Glc (**Figure 3A**). Apart from Gal and Fuc, which were significantly more abundant in B73 than in EA2024, monosaccharide composition did not differ between the two inbreds in non-inoculated internodes. No substantial changes were observed after *F. graminearum* infection in either genotype. Likewise, neither Ara (Ara/AX) nor GlcA (GlcA/AX) substitution levels in arabinoxylans (referred to as Ara + Xyl) were affected across the treatment conditions (**Figures 3C, D**).

### 3.3 Native cell wall polysaccharide analyses obtained by subcritical water extraction

To gain insight into specific cell wall polysaccharide structure in control conditions and upon infection with *F. graminearum*, pith cell walls of B73 and EA2024 were extracted using SWE. Gravimetric yields in these sequential extractions at 10-SWE and 30-SWE fractions were calculated (**Supplementary Figure 5**) and the polysaccharides obtained were subsequently analyzed.

**Figure 4:**
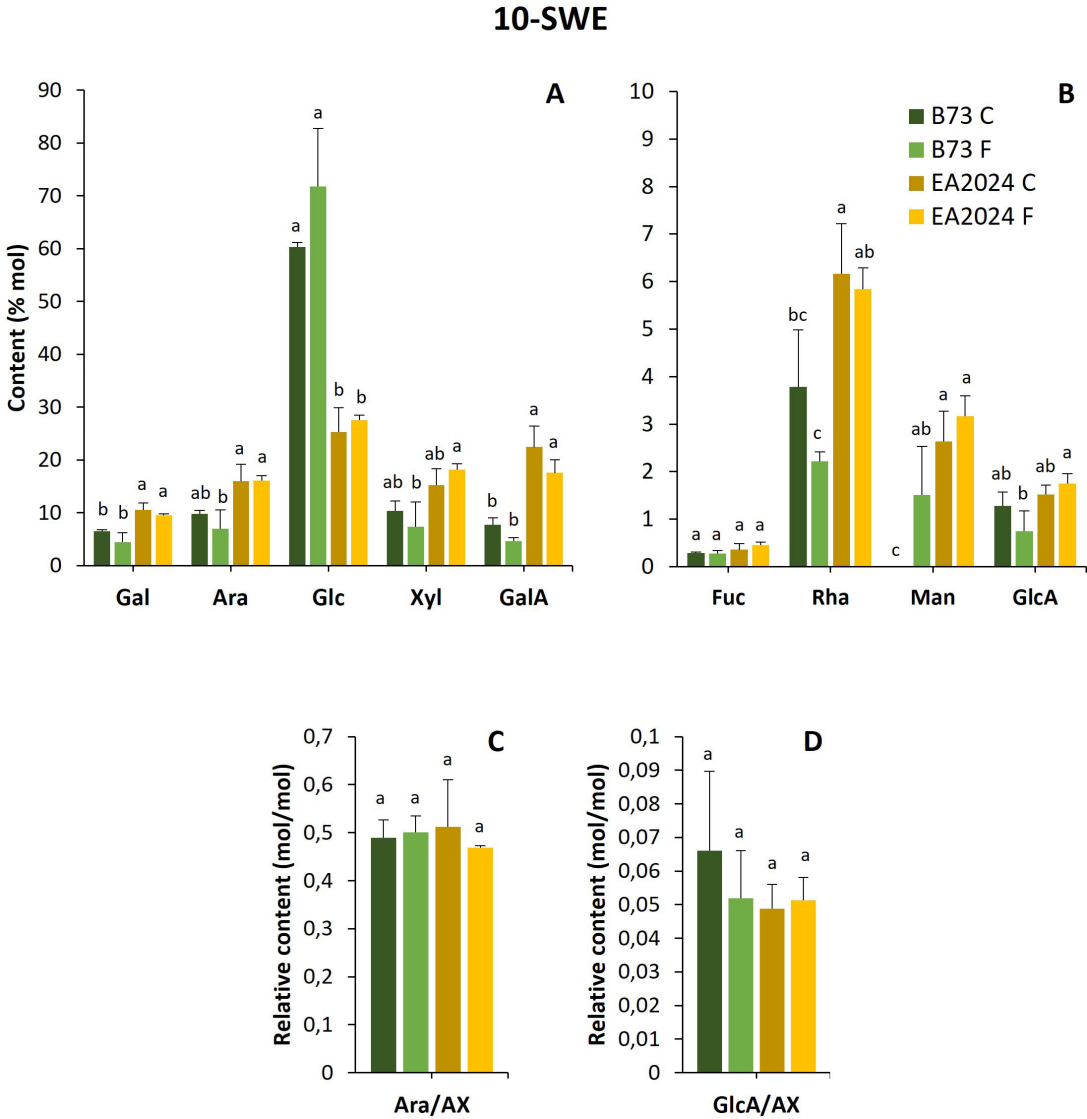
Monosaccharide composition of loosely-bound native polysaccharides obtained by SWE of pith cell walls. **(A, B)** Monosaccharide composition of 10 min fraction (10-SWE) and **(C, D)** molar ratios of substitutions of AX (arabinoxylan referred to as Ara + Xyl) obtained from pith cell walls from B73 and EA2024 maize inbreds grown in control -C- and *F. graminearum -*F- inoculation conditions. Monosaccharides are referred to as: Ara (arabinose), Xyl (xylose), Glc (glucose), Gal (galactose), Rha (rhamnose), Man (mannose), GlcA (glucuronic acid), GalA (galacturonic acid). Data represent x ± SD (n=3). Statistical comparisons were performed independently within, but not between, different compound groups using one-way ANOVA followed by a *post hoc* Tukey test (*p* ≤ 0.05).

**Figure 5:**
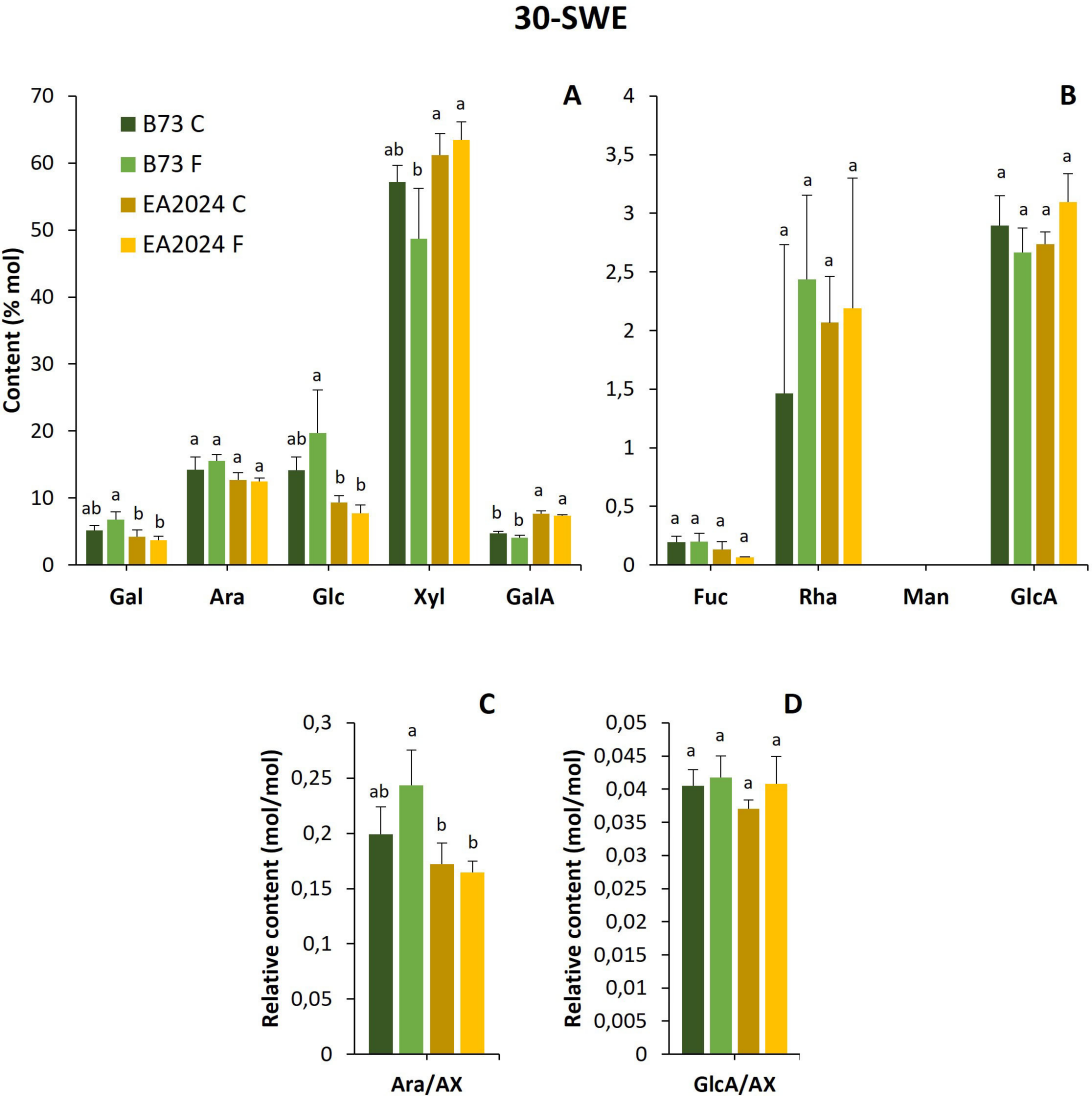
Monosaccharide composition of tightly-bound native polysaccharides obtained by SWE of pith cell walls. **(A, B)** Monosaccharide composition of 30 min fraction (30-SWE) **(C, D)** molar ratios of substitutions of AX (arabinoxylan referred to as Ara + Xyl) from pith cell walls from B73 and EA2024 maize inbreds grown in control -C- and *F. graminearum -*F- inoculation conditions. Monosaccharides are referred to as: Ara (arabinose), Xyl (xylose), Glc (glucose), Gal (galactose), Rha (rhamnose), Man (mannose), GlcA (glucuronic acid), GalA (galacturonic acid). Data represent x ± SD (n=3). Statistical comparisons were performed independently within, but not between, different compound groups using one-way ANOVA followed by a *post hoc* Tukey test (*p* ≤ 0.05).

Since SWE separates polysaccharides according to extractability, the 10-SWE and 30-SWE fractions should be defined as populations enriched in loosely-bound and tightly-bound cell wall polysaccharides. In accordance with this, sequential extractions by SWE resulted in different polysaccharide populations, considering their monosaccharide composition (**Figures 4**, **5**). The 10-SWE fraction contains the loosely-bound polysaccharides and it was mainly composed of Glc (up to 60-70% in B73; **Figure 4A**) followed by Xyl, Ara, GalA and Gal. Comparison between control samples indicated that 10-SWE from EA2024 was significantly enriched in Gal, Rha and GalA compared to B73, suggesting a higher abundance of pectic components, while the proportion of Glc was significantly higher in B73 when compared to EA2024, suggesting an enrichment in non-cellulosic glucan components (i.e. mixed-linkage β-glucans, callose, xyloglucan; **Figures 4A, B**, B73 C vs. EA2024 C) and/or starch (**Supplementary Figure 6**). After infection with *F. graminearum*, only a significant increment in Man content was detected in B73, whereas no variation in other monosaccharide composition of 10-SWE was detected in any of the inbreds analyzed (**Figures 4A, B**). The Ara/AX and GlcA/AX ratios did not show significant differences for either inbred or infection (**Figures 4C, D**).

The 30-SWE fraction contains the tightly-bound polysaccharides, and it was primarily composed of Xyl, which accounted for 45% to 65% of the fraction depending on the sample. The remaining components in order of abundance were Ara, Glc, Gal, and GalA (**Figure 5A**). Variations in the composition of this fraction were observed between control samples, as EA2024 control had significantly higher levels of GalA than those from B73 C (**Figure 5A**, B73 C vs. EA2024 C). No changes were found in the monosaccharide composition of 30-SWE when comparing samples from inoculated plants to their respective controls (**Figures 5A, B**). Regarding Ara/AX and GlcA/AX ratio, no significant differences were observed for either inbred or infection (**Figures 5C, D**).

**Figure 6:**
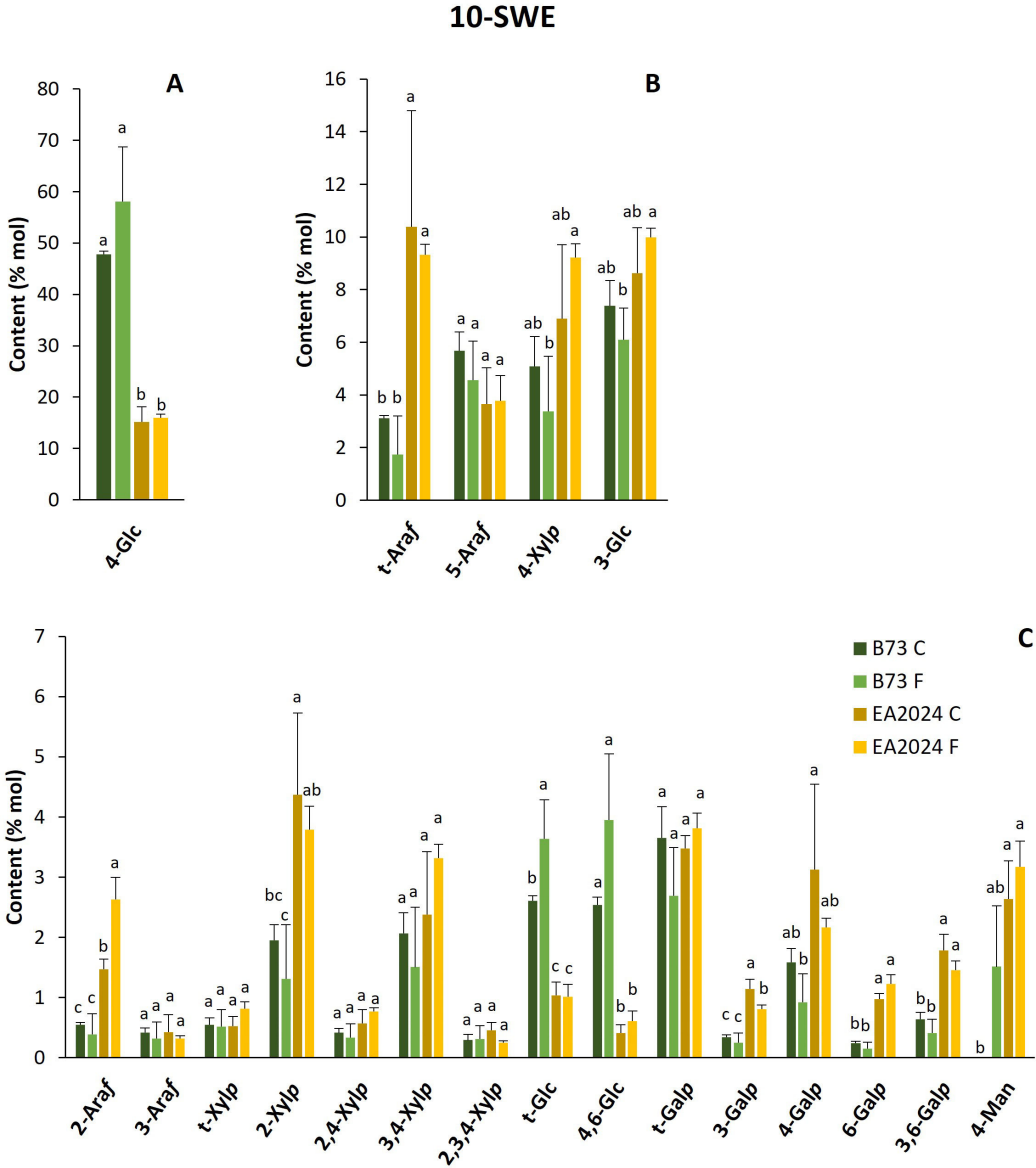
Linkage analysis of loosely-bound native polysaccharides obtained by SWE of pith cell walls. **(A–C)** Glycosidic linkage analysis of 10 min fraction (10-SWE) obtained from pith cell walls from B73 and EA2024 maize inbreds grown in control -C- and *F. graminearum -*F- inoculation conditions. Monosaccharides are referred to as: Ara (arabinose), Xyl (xylose), Glc (glucose), Gal (galactose), Rha (rhamnose), Man (mannose), GlcA (glucuronic acid), GalA (galacturonic acid). Data represent x ± SD (n=3). Statistical comparisons were performed independently within, but not between, different compound groups using one-way ANOVA followed by a *post hoc* Tukey test (*p* ≤ 0.05).

**Figure 7:**
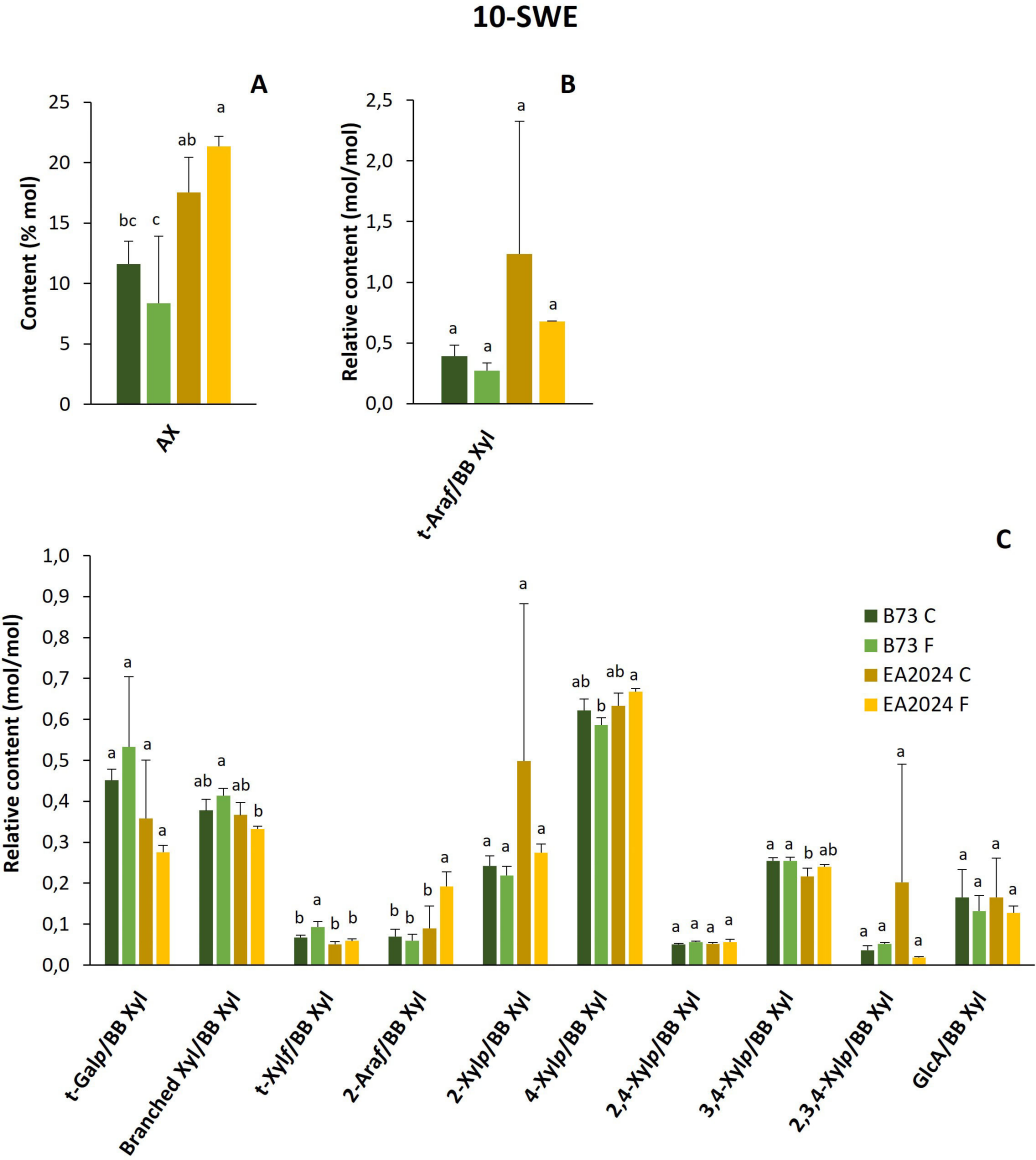
Linkage ratios of loosely-bound native polysaccharides obtained by SWE of pith cell walls. **(A–C)** Glycosidic linkage ratios of 10 min fraction (10-SWE) obtained from pith cell walls from B73 and EA2024 maize inbreds grown in control -C- and *F. graminearum -*F- inoculation conditions. BB means backbone and AX corresponds to arabinoxylans. Monosaccharides are referred to as: Ara (arabinose), Xyl (xylose), Glc (glucose), Gal (galactose), Rha (rhamnose), Man (mannose), GlcA (glucuronic acid), GalA (galacturonic acid). AX content was referred to as the sum of molar percentage of 2-Araf, 3-Araf, t-Xyl, 2-Xylp, 4-Xylp, 2,4- Xylp, 3,4 Xylp and 2,3,4-Xylp. Besides, BB Xyl was calculated considering 4-Xylp, 2,4-Xylp, 3,4-Xylp and 2,3,4-Xylp molar percentages. Data represent x ± SD (n=3). Statistical comparisons were performed independently within, but not between, different compound groups using one-way ANOVA followed by a *post hoc* Tukey test (*p* ≤ 0.05).

**Figure 8:**
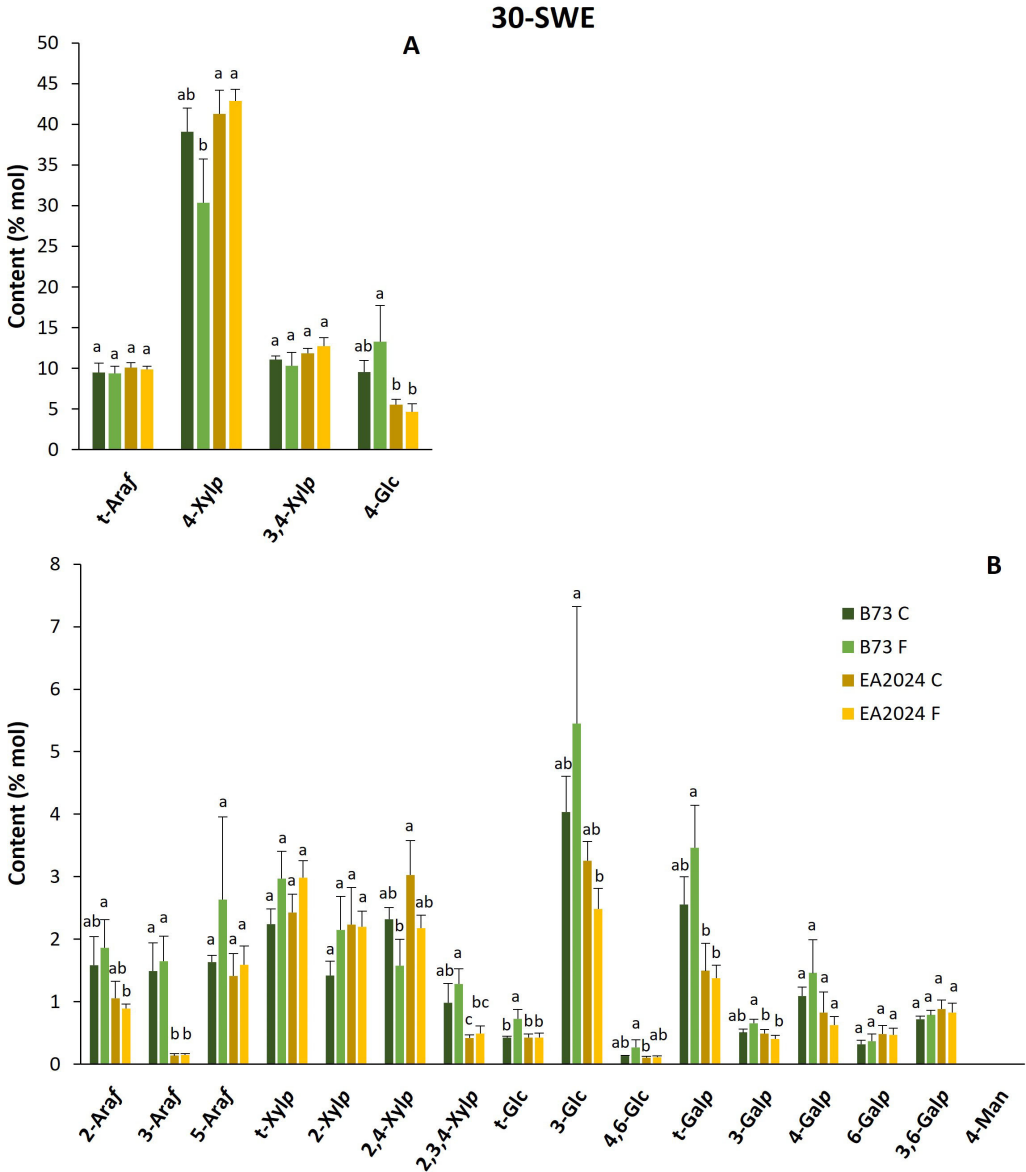
Linkage analysis of tightly-bound native polysaccharides obtained by SWE of pith cell walls. **(A, B)** Glycosidic linkage analysis of 30 min fraction (30-SWE) obtained from pith cell walls from B73 and EA2024 maize inbreds grown in control -C- and *F. graminearum -*F- inoculation conditions. Monosaccharides are referred to as: Ara (arabinose), Xyl (xylose), Glc (glucose), Gal (galactose), Rha (rhamnose), Man (mannose), GlcA (glucuronic acid), GalA (galacturonic acid). Data represent x ± SD (n=3). Statistical comparisons were performed independently within, but not between, different compound groups using one-way ANOVA followed by a *post hoc* Tukey test (*p* ≤ 0.05).

**Figure 9:**
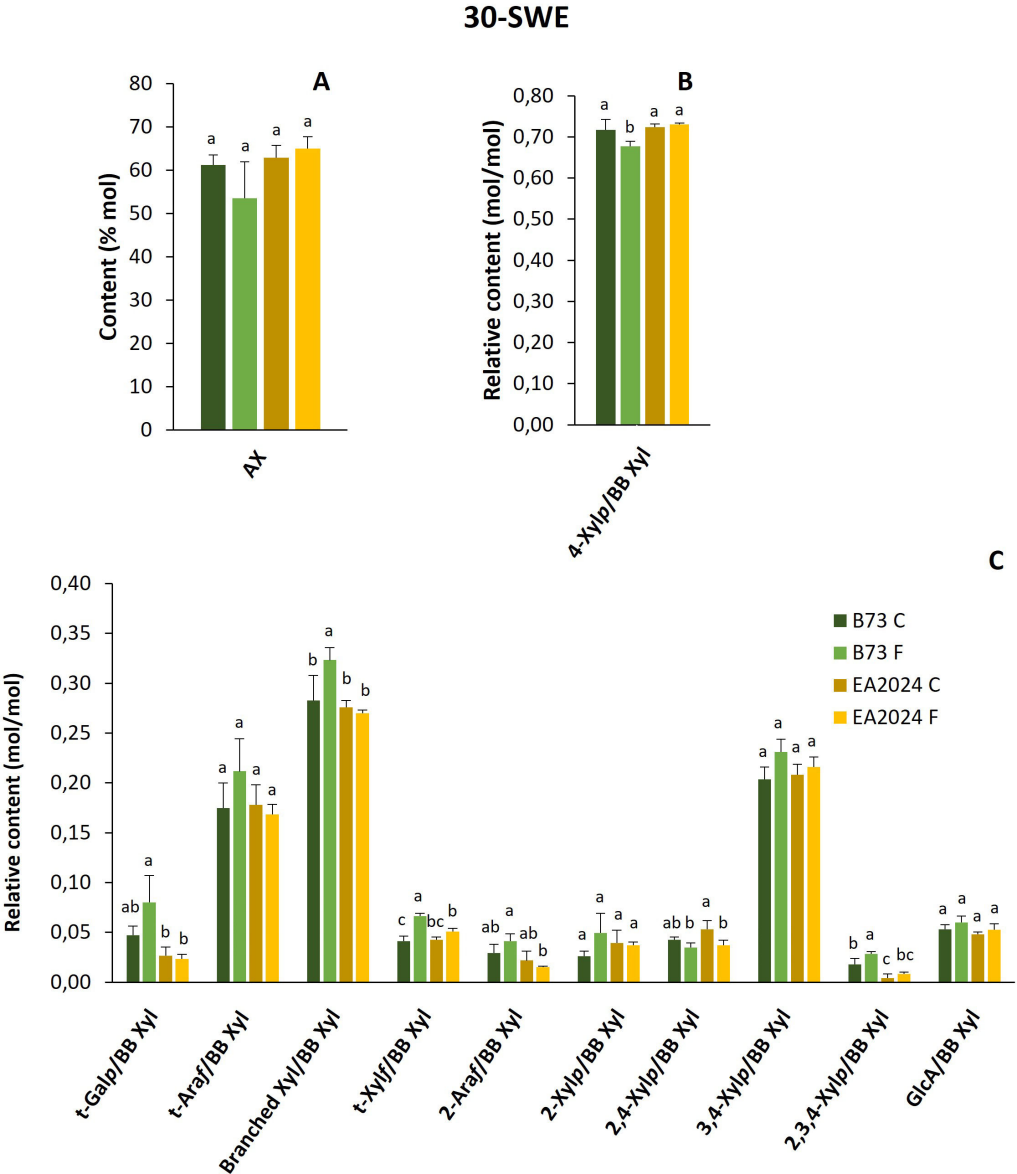
Linkage ratios of tightly-bound native polysaccharides obtained by SWE of pith cell walls. **(A–C)** Glycosidic linkage ratios of 30 min fraction (30-SWE) obtained from pith cell walls from B73 and EA2024 maize inbreds grown in control -C- and *F. graminearum -*F- inoculation conditions. BB means backbone and AX corresponds to arabinoxylans. Monosaccharides are referred to as: Ara (arabinose), Xyl (xylose), Glc (glucose), Gal (galactose), Rha (rhamnose), Man (mannose), GlcA (glucuronic acid), GalA (galacturonic acid). AX content was referred to as the sum of molar percentage of 2-Araf, 3-Araf, t-Xyl, 2-Xylp, 4-Xylp, 2,4- Xylp, 3,4 Xylp and 2,3,4-Xylp. Besides, BB Xyl was calculated considering 4-Xylp, 2,4-Xylp, 3,4-Xylp and 2,3,4-Xylp molar percentages. Data represent x ± SD (n=3). Statistical comparisons were performed independently within, but not between, different compound groups using one-way ANOVA followed by a *post hoc* Tukey test (*p* ≤ 0.05).

Polysaccharides extracted by SWE were subjected to a detailed study by analyzing their glycosidic linkages (**Figure 6**–**9**). The polysaccharidic fraction named 10-SWE was enriched in 4-Glc, t-Araf, 5-Araf, 4-Xylp and 3-Glc (**Figures 6A, B**). Noticeable differences were found mainly with respect to genotypes, not upon inoculation. Specifically, B73 showed significantly higher values for 4-Glc, t-Glc and 4,6-Glc when compared to EA2024. These results are likely to be related to the presence of starch and MLGs (between 11% for B73 C and 7% for B73 F of total SWE-10) in B73 samples (**Supplementary Figure 6**). In EA2024, significantly larger values of t-Araf, 2-Araf, 2-Xylp, 3-Galp, 6-Galp, 3,6-Galp and 4-Man were found when compared to B73 (**Figures 6A, B**). It is particularly interesting that the ratio of 3-Glc versus 4-Glc is very high for EA2024, which could indicate the presence of callose. In addition, an increase in 4-Glc together with 2-Xyl could indicate the presence of more xyloglucan in this line.

Based on the analysis of glycosidic linkages, ratios that provide information about the decoration structure of AXs extracted in 10-SWE were obtained (**Figure 7**). The results indicated that the AXs substitution structure hardly varied between genotypes, as no significant differences in branched Xyl/BB Xyl, 4-Xylp/BB Xyl, 2,4-Xylp/BB Xyl, 2,3,4- Xylp/BB Xyl or GlcA/BB Xyl ratios were found when comparing B73 and EA2024 under control conditions (**Figures 7B, C**). As an exception, the 3,4-Xylp/BB Xyl ratio was significantly lower in EA2024 compared to B73. The study found similar results when comparing control samples to their inoculation treatments. Only the ratio of 2-Araf/BB Xyl showed a significant increase in EA2024 upon inoculation (**Figure 7C**).

The 30-SWE fraction represents the enriched xylan fraction, and it is mainly composed of 4-Xylp followed by t-Araf, 3,4-Xylp and residual 4-Glc (**Figure 8A**). Under control conditions, B73 showed significantly higher contents of 3- Araf and 2,3,4-Xylp compared to EA2024 (**Figures 8A, B**). No significant differences were found upon inoculation, except for t-Glc which significantly increased in B73. In addition, B73 showed approximately 10% of MLGs content in contrast to 4% in EA2024; both inbreds at 30-SWE fraction had around 1% of starch (**Supplementary Figure 6**). The total amount of AXs found at 30-SWE fraction was three times higher than in the 10-SWE fraction. Nevertheless, all the samples displayed similar values among them in these two fractions (**Figure 7A**, **9A**). As previously mentioned, ratios regarding decoration structure of AXs were determined also in 30-SWE fraction (**Figure 9C**). Some differences were observed in the linkages corresponding to the xylan backbone, the terminal and the oligomeric substitutions. Only, significant differences in the ratio 2,3,4-Xylp/BB Xyl between genotypes in control conditions were found, being higher for B73 control (**Figure 9C**). Upon inoculation the 4-Xylp/BB Xyl ratio showed a significant reduction for B73, while for EA2024 remained unchanged (**Figure 9B**). Conversely, significant differences in the ratios of branched Xyl/BB Xyl, t-Xylp/BB Xyl, and 2,3,4-Xylp/BB Xyl were observed only in the case of B73, being higher under infection conditions (**Figure 9C**, B73 C vs. B73 F). In case of 2,4-Xylp/BB Xyl ratio, EA2024 inoculated plants presented significantly lower values when compared to its control (**Figure 9C**).

## 4 Discussion

Most maize inbreds and hybrids are susceptible to *Fusarium* species (Kikot et al., 2009; Mesterházy et al., 2012; Quesada-Ocampo et al., 2016), leading to significant economic losses. Therefore, it is crucial to determine the traits underlying *Fusarium* resistance, as they may be used as target in breeding programs. Particularly, the composition and structure of plant cell wall condition the susceptibility to CWDEs produced by pathogens during infection processes, affecting host-pathogen interactions (Lionetti et al., 2015). Thus, in the present study, the resistance to *F. graminearum* of two maize inbred lines (B73 and EA2024) was evaluated, followed by a detailed biochemical characterization of their cell wall composition. Although B73 was previously classified as lodging-resistant based on mechanical stalk strength, our results indicate that lodging resistance does not necessarily predict resistance to *F. graminearum*. These traits likely depend on partially different structural determinants. In our study, fungal resistance was evaluated in pith tissue, where EA2024 displayed cell wall features potentially more favorable for defense, despite its lower lodging resistance.

### 4.1 B73 and EA2024 genotypes present phenotypic and index damage differences upon inoculation

As fungus *F. graminearum* causes extensive stalk rot disease in maize (Brauer et al., 2020; Zhang et al., 2016; Zuo et al., 2022), the inoculation of a spore suspension was performed directly in the stalks of the two inbreds (B73 and EA2024). The hyphae of *F. graminearum*, which is a hemibiotrophic fungus, develops intercellularly at early points (biotrophic phase), while after 72 h post-infection, pith parenchyma cells are invaded and brown lesions are noticeable (necrotrophic phase; Zhang et al., 2016). The visible disease symptoms showed significant differences between B73 and EA2024 infected plants, being widespread at 25 dpi in both trials (**Figures 1A, B**). This visual evaluation indicated that the speed of progression of *F. graminearum* through the pith was higher for B73 in comparison to EA2024. In addition, the presence of a conserved fragment of 16N sequence in the internode used for cell wall analysis was tested (**Supplementary Figure 2**). The results indicated that although there may be minor cross contamination in the control material, since this is a field experiment, only the B73 plants at 25 dpi showed a noticeable presence of the fungus. In summary, the two genotypes showed phenotypic differences upon inoculation, being B73 susceptible to *F. graminearum* which agrees with previous studies (Pechanova et al., 2013; Quesada-Ocampo et al., 2016; Su et al., 2025).

### 4.2 Differences in minor cell wall components were detected in maize inbred lines with contrasting resistance against *F. graminearum*

As indicated before, cell wall composition and structure are key factors affecting plant-pathogen interactions (Lionetti et al., 2015). Possible cell wall changes through the infection process were studied using two different responsive genotypes (susceptible -B73- and resistant -EA2024). For this purpose, cell walls of the pith tissue of the second internode below the main ear (an internode different from the one inoculated) were analyzed. Although the fungus was molecularly detected in the analyzed internode (**Supplementary Figure 2**), no severely damaged tissue was observed in any of the samples analyzed. During infection, the damage becomes visible when *F. graminearum* causes tissue maceration. However, by that point, the maize cell walls have undergone significant changes due to the CWDEs released by the fungus, making it impossible to determine the structural changes the plant has induced as a defense mechanism. This is the reason why in this study the cell wall analyses were performed in an internode different from the inoculated one, where no severely damaged tissue was observed. Accordingly, these data mainly reflect constitutive differences between genotypes rather than extensive local remodeling in severely infected tissue. In contrast, infection-induced effects in the analyzed tissue were limited, with only few changes detected, particularly in B73.

The level of DFAT in the pith cell walls may play a role in the early infection process of *F. graminearum* in maize (Santiago et al., 2007). The results presented here indicate that *p*CA was the most abundant cell wall hydroxycinnamate, followed by FA and minor amounts of DFAT, in agreement with the already reported in maize pith tissues (Santiago et al., 2007). However, in the current study, the profile of cell wall ester-bound phenolics differed between B73 and EA2024 but remained unchanged after inoculation (**Figure 2**). Our data suggests that the constitutive diferulate composition may explain, rather than infection-induced changes, differences in susceptibility to *F. graminearum* in these genotypes. In this context, no clear conclusions are found related to the role of diferulates in *F. graminearum* stalk rot resistance. It is worth noting that our inoculation method involves direct injection into the pith tissue, and it is possible that diferulates may play a more significant role in the reinforcement of the cortex during the natural progress of the fungus during infection. Nevertheless, the increased level of *p*CA in EA2024 may suggest a role for this compound in *F. graminearum* resistance. Although the lignin content did not vary, a slight increasing trend was observed in EA2024 after infection (**Supplementary Figure 3C**). Due to the positive relationship between *p*CA and cell wall lignification already described (Hatfield and Marita, 2010), this fact may contribute to resistance. Previous studies have shown that a high level of *p*CA bound to the cell wall could be indirectly linked to insect resistance through its association with S-subunits of the lignin polymer (Gesteiro et al., 2021). This highlights the importance of lignin composition rather than its content.

MLGs are present in the cell wall of monocotyledonous plants such as maize but are also abundant in bacterial and fungal species. MLGs play multifaceted roles in the primary cell walls, acting as structural components during growth, reinforcing mature tissues, and serving as temporary energy storage (Kim and Brandizzi, 2021). Unlike other hemicelluloses, MLGs are unbranched polymers that provide flexibility and strength, helping to manage cell wall integrity. In this regard, B73, a line previously classified by our group as resistant to lodging (Manga-Robles et al., 2021), had twice as many MLGs in its cell walls as EA2024 (**Figure 2D**), a line classified as susceptible to lodging, suggesting that this polysaccharide could play a structural role. In addition, MLGs have been described as potential defense elicitors in monocots and dicots (Rebaque et al., 2021; Barghahn et al., 2021). However, the MLG content did not change in either inbred after infection (**Figure 2D**). However, our measurements only quantify total MLG content and do not assess MLG-derived degradation products or their signaling activity. Therefore, we cannot rule out a role for MLG-derived DAMPs in this pathosystem. Our results only indicate that total MLG abundance did not change after infection in the tissue analyzed. Nevertheless, it must be considered that the internode studied did not show any visible symptoms of degradation.

On the other hand, a high constitutive presence of 3-Glc relative to 4-Glc in EA2024 could be associated not only with MLG but also with callose deposits. This increased callose presence in the resistant line may represent a defensive reinforcement in response to the incipient presence of *F. graminearum*, even in the absence of visual symptoms.

### 4.3 Pectin population may participate in the defense against *Fusarium graminearum* infection

The following sequential extractions carried out by SWE provide insight into the extractability of the different polysaccharides found in these cell walls. In cereals and specifically in maize, pectins, glucans and starch are expected to be extracted at earlier times, leading to a progressive enrichment in glucuronoarabinoxylan (GAX) at longer extraction times (Rudjito et al., 2019; Ruthes et al., 2020). Our results agree with these previous studies since pectin-related monosaccharides were extracted at an early time (10-SWE, **Figure 4**; Couture et al., 2021; Ruthes et al., 2020). Addressing the differences between inbreds, GalA, Rha, Gal and Ara were found at higher levels in case of EA2024 (**Figure 3A**), thus suggesting a higher pectin content in this inbred. In line with this, GalA content in cell wall, although nonsignificant, showed a trend to be higher for EA2024 (**Figure 3B**). It is known that GalA, the main uronic acid constituent of pectins, showed a significant negative correlation with the incidence and severity of *F. graminearum* in wheat (Giancaspro et al., 2018), which agrees with the lower fungus-induced damage found in EA2024 when compared to B73. In contrast, B73 was characterized by a lower content of pectin-related monosaccharides together with an increased level of Glc compared to EA2024. This increased Glc content for B73 may be related to the presence of starch (**Supplementary Figure 3E**) and MLGs (**Figure 2D**), which accounted for 22%-35% and 6%-12%, respectively (B73 C vs. B73 F; Rudjito et al., 2019).

As mentioned above, EA2024 10-SWE fraction had an elevated content of GalA (**Figure 4**), which may suggest a higher level of homogalacturonan. In addition, this GalA linked with Rha, which is also found in higher concentrations, suggests the presence of rhamnogalacturonan-I, and their corresponding decorations, such as arabinan (Ara content), galactan (Gal content) and arabinogalactan side chains (Gal content) (**Figure 4**). Besides, some of the neutral sugars found by glycosidic analysis may confirm the hypothesis suggested by monosaccharide determinations. EA2024 showed higher 4-Galp which can be originated from galactan-side chains of rhamnogalacturonan-I (**Figure 6**). Furthermore, this inbred showed increased levels of 6-Galp, 3, 6-Galp, 3-Galp and t-Ara, which may suggest the presence of arabinogalactan. The higher amount of 4-Man found in EA2024 may reveal the presence of mannans in maize cell walls as previously reported (Couture et al., 2021; Rudjito et al., 2019), which are also a reservoir of DAMPs (Zang et al., 2019). In case of B73 linkage analysis, 10-SWE fraction was enriched in 4-Glc, 4, 6-Glc and t-Glc, which may confirm the increased presence of starch in this inbred (Rudjito et al., 2019).

During *Fusarium* infection, pectinases such as polygalacturonase and pectate lyase are the first produced, becoming determinant in the development of the disease (Aleandri et al., 2007; Ferrari et al., 2012; Kikot et al., 2009; Niture et al., 2006; Tomassini et al., 2009). Although pectins are a minor component in maize mature stalks, they may be relevant in the infection process. *F. graminearum* produces high amounts of pectic hydrolytic enzymes during infection (Kikot et al., 2009). Plants can recognize these degradation products (mono- or oligosaccharides) as DAMPs (Lionetti et al., 2015). Pectin-derived oligogalacturonides produced by fungal pectinase activity are well-known DAMPs (De Azevedo Souza et al., 2017; Ferrari et al., 2013; Swaminathan et al., 2022; Voxeur and Höfte, 2020). These elicitors activate innate plant defense through signal transduction and transcription activation (Gille and Pauly, 2012), allowing different strategies depending on the genotype. Resistant genotypes prioritize the activation of early defense responses, whereas susceptible lines undergo pathogen-favoring metabolic reprogramming (Su et al., 2025). Although no quantitative changes in pectin content have been detected due to infection in our experiment, it should be noted that the internode analyzed it upper positioned and did not show symptoms of active cell wall degradation.

Taken together, the higher abundance of pectin-related polysaccharides in EA2024 suggests a cell wall composition potentially associated with its higher resistance to *F. graminearum*. However, as DAMP release and immune activation were not directly measured in this study, this interpretation should be considered hypothetical.

### 4.4 Changes in arabinoxylan substitutions may impact maize resistance to *F. graminearum*

The substantial increase from 10-SWE to 30-SWE of 4-Xylp BB and Xyl in monosaccharide analyses revealed a xylan-enriched population at longer times. Linkage analysis of AX allows us to indicate that, in terms of molar percentage, differences in AX structure were genotype-dependent and that no significant changes were found in either inbred upon inoculation (**Figure 7**). However, in case of the ratios calculated to find out possible decorations of AXs, B73 showed some interesting differences with respect to EA2024, and also in the case of B73 infected vs. B73 control (**Figure 8**). Although double-substituted Xyl are scarce in AXs of the 30-SWE (2,3,4-Xylp/BB Xyl), B73 showed significantly higher levels with respect to EA2024 in control conditions. In summary, these data would point to a remodeling of AX in B73 due to infection, which essentially consisted of an increase in highly substituted AXs and the possible presence of oligosaccharide side chains. This assumption is supported by the data presented in **Figures 8B**, **5C**, which show: 1) a significant increase in branched Xyl/BB Xyl and specifically reflected in the 2,3,4-Xylp/BB Xyl; 2) a decrease in unsubstituted Xyl (4-Xylp/BB Xyl); 3) an increase in the ratios for BB Xyl regarding t-Galp, t-Xylf, 2-Araf and 2-Xylp; 4) a trend to be more Ara-substituted as indicated by a higher Ara/AX (**Figures 5C**, **8C**). These changes may be produced by B73, susceptible inbred, to try to overcome the infection. However, these double and larger AX substitutions may impact this process negatively. It is known that highly substituted GAXs play a filler role in the interstitial matrix, while the less substituted GAXs are tightly associated with cellulose microfibrils, enhancing cell wall strength (Carpita et al., 2001; Kozlova et al., 2014; Tabuchi et al., 2011; Tryfona et al., 2023; Wang et al., 2014, Wang et al., 2016). Altogether, the higher disubstituted Xyl/BB Xyl in B73 may affect the interaction with other cell wall components (Tryfona et al., 2023), resulting in easily digestible cell walls by the fungus. Besides, during the infection process, B73 increases double substitutions in Xyls and oligosaccharidic side chains of GAXs, which may result in fewer or weaker polysaccharide interconnections, favoring the spread of the fungus. These results indicate that infection in B73 is associated with changes in arabinoxylan substitution patterns. Although such changes may affect polysaccharide interactions within the cell wall, we did not directly assess cell wall mechanics or fungal spread dynamics; therefore, any functional consequence should be regarded as a hypothesis.

## 5 Conclusions

Our results highlight the complexity of the maize–*Fusarium* pathosystem and support the idea that stalk cell wall composition is associated with contrasting levels of resistance in inbreds B73 and EA2024, which may determine the outcome of *F. graminearum* infection under naturally changing environmental conditions. In this field assay, we have shown that inbred lines B73 and EA2024 exhibit markedly different levels of resistance, associated with their constitutive cell wall architecture.

Under our experimental conditions, EA2024 showed a higher abundance of loosely-bound polysaccharides, including pectin-related fractions, mannans, and putative callose-associated features, whereas B73 showed a higher proportion of disubstituted xylans in the tightly-bound fraction. These differences may be relevant to the contrasting responses of both genotypes to *F. graminearum*. However, further functional analyses will be required to determine whether these traits contribute directly to defense or fungal spread.

Importantly, infection-induced remodeling in B73—evidenced by increased disubstitution and oligomeric side chains in arabinoxylans—appears to be an ineffective defense strategy. This structural reconfiguration likely results in a less cohesive and more accessible cell wall matrix, ultimately promoting fungal colonization. Although higher levels of ferulic acid, diferulic acid, and mixed-linkage glucans in B73 may contribute to an enhanced mechanical strength as described in Manga-Robles et al. (2021), these features are insufficient to prevent fungal penetration across the pith. However, it should be noted that these findings require cautious interpretation. As the analyses were carried out in an internode different from the inoculated one, where no severely damaged tissue was observed, the data primarily reflect constitutive genotype-dependent wall traits and only limited infection-associated remodeling in the analyzed tissue. In this context, the changes observed in arabinoxylan substitution patterns, particularly in B73, may be associated with susceptibility. However, further research is needed to establish their functional significance.

Taken together, our findings underscore that both genotype and subtle modifications in minor components as well as substitutions within major polysaccharides(arabinoxylans), play a role in modulating cell wall resistance to pathogen attack. Given the quantitative and multifactorial nature of disease resistance under field conditions, these insights are highly relevant for advancing our understanding of maize stalk rot and, more importantly, for identifying novel cell wall–based molecular markers to support the development of *Fusarium*-resistant maize genotypes.

## Data Availability

The raw data supporting the conclusions of this article will be made available by the authors, without undue reservation.

## References

[B1] Aleandri M. P. Magro P. Chilosi G. (2007). Modulation of host pH during the wheat–Fusarium culmorum interaction and its influence on the production and activity of pectolytic enzymes. Plant Pathol. 56, 517–525. doi:10.1111/j.1365-3059.2007.01574.x 40046247

[B2] An H. J. Lurie S. Greve L. C. Rosenquist D. Kirmiz C. Labavitch J. M. . (2005). Determination of pathogen-related enzyme action by mass spectrometry analysis of pectin breakdown products of plant cell walls. Anal. Biochem. 338, 71–82. doi:10.1016/j.ab.2004.11.004 15707937

[B3] Barghahn S. Arnal G. Jain N. Petutschnig E. Brumer H. Lipka V. (2021). Mixed linkage β-1,3/1,4-glucan oligosaccharides induce defense responses in Hordeum vulgare and Arabidopsis thaliana. Front. Plant Sci. 12, 682439. doi:10.3389/fpls.2021.682439 34220903 PMC8247929

[B4] Brauer E. K. Subramaniam R. Harris L. J. (2020). Regulation and dynamics of gene expression during the life cycle of Fusarium graminearum. Phytopathology 110, 1368–1374. doi:10.1094/PHYTO-03-20-0080-IA 32460691

[B5] Broders K. D. Lipps P. E. Paul P. A. Dorrance A. E. (2007). Evaluation of Fusarium graminearum associated with corn and soybean seed and seedling disease in Ohio. Plant Dis. 91, 1155–1160. doi:10.1094/PDIS-91-9-1155 30780657

[B6] Brown N. A. Evans J. Mead A. Hammond-Kosack K. E. (2017). A spatial temporal analysis of the Fusarium graminearum transcriptome during symptomless and symptomatic wheat infection. Mol. Plant Pathol. 18, 1295–1312. doi:10.1111/mpp.12564 28466509 PMC5697668

[B7] Cacique I. S. Pinto L. F. C. C. Aucique-Pérez C. E. Wordell Filho J. A. Rodrigues F. A. (2020). Physiological and biochemical insights into the basal level of resistance of two maize hybrids in response to Fusarium verticillioides infection. Plant Physiol. Biochem. 152, 194–210. doi:10.1016/j.plaphy.2020.04.036 32422536

[B8] Calderone S. Mauri N. Manga-Robles A. Fornalé S. García-Mir L. Centeno M.-L. . (2024). Diverging cell wall strategies for drought adaptation in two maize inbreds with contrasting lodging resistance. Plant Cell Environ. 47, 1747–1768. doi:10.1111/pce.14822 38317308

[B9] Cantu D. Vicente A. R. Labavitch J. M. Bennett A. B. Powell A. L. T. (2008). Strangers in the matrix: plant cell walls and pathogen susceptibility. Trends Plant Sci. 13, 610–617. doi:10.1016/j.tplants.2008.09.002 18824396

[B10] Carpita N. C. Defernez M. Findlay K. Wells B. Shoue D. A. Catchpole G. . (2001). Cell wall architecture of the elongating maize coleoptile. Plant Physiol. 127, 551–565. doi:10.1104/pp.010146 11598229 PMC125090

[B11] Couture G. Vo T.-T. T. Castillo J. J. Mills D. A. German J. B. Maverakis E. . (2021). Glycomic mapping of the maize plant points to greater utilization of the entire plant. ACS Food Sci. Technol. 1, 2117–2126. doi:10.1021/acsfoodscitech.1c00318

[B12] De Azevedo Souza C. A. Li S. Lin A. Z. Boutrot F. Grossmann G. Zipfel C. . (2017). Cellulose-derived oligomers act as damage-associated molecular patterns and trigger defense-like responses. Plant Physiol. 173, 2383–2398. doi:10.1104/pp.16.01680 28242654 PMC5373054

[B13] Dence C. W. (1992). “ The determination of lignin,” in Methods in lignin chemistry. Eds. Lin S. Y. Dence C. W. ( Springer, Berlin), 33–61. doi:10.1007/978-3-642-74065-7_3

[B14] Dische Z. (1962). “ Color reactions of pentoses,” in Methods in carbohydrate chemistry. Eds. Whistler R. L. Wolfrom M. L. ( Academic Press, New York), 475–514.

[B15] Engelsdorf T. Hamann T. (2014). An update on receptor-like kinase involvement in the maintenance of plant cell wall integrity. Ann. Bot. 114, 1339–1347. doi:10.1093/aob/mcu043 24723447 PMC4195549

[B16] Ferrari S. Savatin D. V. Sicilia F. Gramegna G. Cervone F. De Lorenzo G. (2013). Oligogalacturonides: plant damage-associated molecular patterns and regulators of growth and development. Front. Plant Sci. 4, 49. doi:10.3389/fpls.2013.00049 23493833 PMC3595604

[B17] Ferrari S. Sella L. Janni M. De Lorenzo G. Favaron F. D’Ovidio R. (2012). Transgenic expression of polygalacturonase-inhibiting proteins in Arabidopsis and wheat increases resistance to the flower pathogen Fusarium graminearum. Plant Biol. (Stuttg) 14, 31–38. doi:10.1111/j.1438-8677.2011.00449.x 21974721

[B18] Gai X. T. Xuan Y. H. Gao Z. G. (2017). Diversity and pathogenicity of Fusarium graminearum species complex from maize stalk and ear rot strains in northeast China. Plant Pathol. 66, 1267–1275. doi:10.1111/ppa.12670 40046247

[B19] García-Olmedo F. Rodríguez-Palenzuela P. Molina A. Alamillo J. M. López-Solanilla E. Berrocal-Lobo M. . (2001). Antibiotic activities of peptides, hydrogen peroxide and peroxynitrite in plant defence. FEBS Lett. 498, 219–222. doi:10.1016/s0014-5793(01)02456-5 11412861

[B20] Gesteiro N. Butrón A. Estévez S. Santiago R. (2021). Unraveling the role of maize (Zea mays L.) cell-wall phenylpropanoids in stem-borer resistance. Phytochemistry 185, 112683. doi:10.1016/j.phytochem.2021.112683 33582589

[B21] Giancaspro A. Lionetti V. Giove S. L. Zito D. Fabri E. Reem N. . (2018). Cell wall features transferred from common into durum wheat to improve Fusarium Head Blight resistance. Plant Sci. 274, 121–128. doi:10.1016/j.plantsci.2018.05.016 30080595

[B22] Gille S. Pauly M. (2012). O-Acetylation of plant cell wall polysaccharides. Front. Plant Sci. 3, 12. doi:10.3389/fpls.2012.00012 22639638 PMC3355586

[B23] Gkolemis K. Giannoutsou E. Adamakis I.-D. S. Galatis B. Apostolakos P. (2023). Cell wall anisotropy plays a key role in Zea mays stomatal complex movement: the possible role of the cell wall matrix. Plant Mol. Biol. 113, 331–351. doi:10.1007/s11103-023-01393-x 38108950 PMC10730690

[B24] Hansey C. N. Lorenz A. J. de Leon N. (2010). Cell wall composition and ruminant digestibility of various maize tissues across development. Bioenerg. Res. 3, 28–37. doi:10.1007/s12155-009-9068-4 30311153

[B25] Hatfield R. D. Marita J. M. (2010). Enzymatic processes involved in the incorporation of hydroxycinnamates into grass cell walls. Phytochem. Rev. 9, 35–45. doi:10.1007/s11101-010-9165-1 30311153

[B26] Houston K. Tucker M. R. Chowdhury J. Shirley N. Little A. (2016). The plant cell wall: a complex and dynamic structure as revealed by the responses of genes under stress conditions. Front. Plant Sci. 7, 984. doi:10.3389/fpls.2016.00984 27559336 PMC4978735

[B27] Jackson-Ziems T. Rees J. Harveson R. M. (2014). Common stalk rot diseases of corn. Department Plant Pathology: Faculty Publications. Extension Circular 1898 (EC1898), University of Nebraska–Lincoln.

[B28] Jedidi I. Jurado M. Cruz A. Trabelsi M. M. Said S. González-Jaén M. T. (2021). Phylogenetic analysis and growth profiles of Fusarium incarnatum-equiseti species complex strains isolated from Tunisian cereals. Int. J. Food Microbiol. 353, 109297. doi:10.1016/j.ijfoodmicro.2021.109297 34153829

[B29] Juroszek P. Von Tiedemann A. (2013). Climatic changes and the potential future importance of maize diseases: a short review. J. Plant Dis. Prot. 120, 49–56. doi:10.1007/BF03356454 30311153

[B30] Kikot G. E. Hours R. A. Alconada T. M. (2009). Contribution of cell wall degrading enzymes to pathogenesis of Fusarium graminearum: a review. J. Basic Microbiol. 49, 231–241. doi:10.1002/jobm.200800231 19025875

[B31] Kim S.-J. Brandizzi F. (2021). Advances in cell wall matrix research with a focus on mixed-linkage glucan. Plant Cell Physiol. 62, 1839–1846. doi:10.1093/pcp/pcab106 34245308

[B33] Kozlova L. V. Ageeva M. V. Ibragimova N. N. Gorshkova T. A. (2014). Arrangement of mixed-linkage glucan and glucuronoarabinoxylan in the cell walls of growing maize roots. Ann. Bot. 114, 1135–1145. doi:10.1093/aob/mcu125 25086589 PMC4195558

[B32] Kozlova L. V. Nazipova A. R. Gorshkov O. V. Petrova A. A. Gorshkova T. A. (2020). Elongating maize root: zone-specific combinations of polysaccharides from type I and type II primary cell walls. Sci. Rep. 10, 10956. doi:10.1038/s41598-020-67782-0 32616810 PMC7331734

[B34] Lionetti V. Giancaspro A. Fabri E. Giove S. L. Reem N. Zabotina O. A. . (2015). Cell wall traits as potential resources to improve resistance of durum wheat against Fusarium graminearum. BMC Plant Biol. 15, 6. doi:10.1186/s12870-014-0369-1 25597920 PMC4298115

[B35] Manga-Robles A. Santiago R. Malvar R. A. Moreno-González V. Fornalé S. López I. . (2021). Elucidating compositional factors of maize cell walls contributing to stalk strength and lodging resistance. Plant Sci. 307, 110882. doi:10.1016/j.plantsci.2021.110882 33902850

[B36] Manoli A. Sturaro A. Trevisan S. Quaggiotti S. Nonis A. (2012). Evaluation of candidate reference genes for qPCR in maize. J. Plant Physiol. 169, 807–815. doi:10.1016/j.jplph.2012.01.019 22459324

[B37] Martínez-Abad A. Giummarella N. Lawoko M. Vilaplana F. (2018). Differences in extractability under subcritical water reveal interconnected hemicellulose and lignin recalcitrance in birch hardwoods. Green Chem. 20, 2534–2546. doi:10.1039/C8GC00385H

[B38] Martínez-Abad A. Jiménez-Quero A. Wohlert J. Vilaplana F. (2020). Influence of the molecular motifs of mannan and xylan populations on their recalcitrance and organization in spruce softwoods. Green Chem. 22, 3956–3970. doi:10.1039/D0GC01207F

[B39] Mesterházy Á. Lemmens M. Reid L. M. (2012). Breeding for resistance to ear rots caused by Fusarium spp. in maize – a review. Plant Breed. 131, 1–19. doi:10.1111/j.1439-0523.2011.01936.x 40046247

[B40] Molina A. Miedes E. Bacete L. Rodríguez T. Mélida H. Denancé N. . (2021). Arabidopsis cell wall composition determines disease resistance specificity and fitness. Proc. Natl. Acad. Sci. 118, e2010243118. doi:10.1073/pnas.2010243118 33509925 PMC7865177

[B41] Niture S. K. Kumar A. R. Pant A. (2006). Role of glucose in production and repression of polygalacturonase and pectate lyase from phytopathogenic fungus Fusarium moniliforme NCIM 1276. World J. Microbiol. Biotechnol. 22, 893–899. doi:10.1007/s11274-006-9119-3 30311153

[B42] Pechanova O. Takáč T. Samaj J. Pechan T. (2013). Maize proteomics: an insight into the biology of an important cereal crop. Proteomics 13, 637–662. doi:10.1002/pmic.201200275 23197376

[B43] Pestka J. J. (2010). Deoxynivalenol: mechanisms of action, human exposure, and toxicological relevance. Arch. Toxicol. 84, 663–679. doi:10.1007/s00204-010-0579-8 20798930

[B44] Pettolino F. A. Walsh C. Fincher G. B. Bacic A. (2012). Determining the polysaccharide composition of plant cell walls. Nat. Protoc. 7, 1590–1607. doi:10.1038/nprot.2012.081 22864200

[B45] Quesada-Ocampo L. M. Al-Haddad J. Scruggs A. C. Buell C. R. Trail F. (2016). Susceptibility of maize to stalk rot caused by Fusarium graminearum deoxynivalenol and zearalenone mutants. Phytopathology 106, 920–927. doi:10.1094/PHYTO-09-15-0199-R 27050573

[B46] Rampersad S. N. (2020). Pathogenomics and management of Fusarium diseases in plants. Pathogens 9, 340. doi:10.3390/pathogens9050340 32369942 PMC7281180

[B48] Rebaque D. Del Hierro I. López G. Bacete L. Vilaplana F. Dallabernardina P. . (2021). Cell wall-derived mixed-linked β-1,3/1,4-glucans trigger immune responses and disease resistance in plants. Plant J. 106, 601–615. doi:10.1111/tpj.15185 33544927 PMC8252745

[B47] Rebaque D. Martínez-Rubio R. Fornalé S. García-Angulo P. Alonso-Simón A. Álvarez J. M. . (2017). Characterization of structural cell wall polysaccharides in cattail (Typha latifolia): Evaluation as potential biofuel feedstock. Carbohydr. Polym. 175, 679–688. doi:10.1016/j.carbpol.2017.08.021 28917917

[B49] Rincón E. Espinosa E. García-Domínguez M. T. Balu A. M. Vilaplana F. Serrano L. . (2021). Bioactive pectic polysaccharides from bay tree pruning waste: Sequential subcritical water extraction and application in active food packaging. Carbohydr. Polym. 272, 118477. doi:10.1016/j.carbpol.2021.118477 34420736

[B51] Rudjito R. C. Jiménez-Quero A. Hamzaoui M. Kohnen S. Vilaplana F. (2020). Tuning the molar mass and substitution pattern of complex xylans from corn fibre using subcritical water extraction. Green Chem. 22, 8337–8352. doi:10.1039/D0GC02897E

[B50] Rudjito R. C. Ruthes A. C. Jiménez-Quero A. Vilaplana F. (2019). Feruloylated arabinoxylans from wheat bran: optimization of extraction process and validation at pilot scale. ACS Sustain. Chem. Eng. 7, 13167–13177. doi:10.1021/acssuschemeng.9b02329

[B52] Ruthes A. C. Rudjito R. C. Rencoret J. Gutiérrez A. del Río J. C. Jiménez-Quero A. . (2020). Comparative recalcitrance and extractability of cell wall polysaccharides from cereal (wheat, rye, and barley) brans using subcritical water. ACS Sustain. Chem. Eng. 8, 7192–7204. doi:10.1021/acssuschemeng.0c01764

[B53] Saeman J. F. Moore W. E. Millett M. A. (1963). “ Sugar units present,” in Cellulose: hydrolysis and quantitative paper chromatography. Ed. W.R.L. ( Academic Press, New York), 54–69.

[B54] Salcedo A. Al-Haddad J. Buell C. R. Trail F. Góngora-Castillo E. Quesada-Ocampo L. (2021). Comparative transcriptome analysis of two contrasting maize inbred lines provides insights on molecular mechanisms of stalk rot resistance. PhytoFrontiers 1(4), 314–329. doi:10.1094/PHYTOFR-12-20-0055-R

[B55] Santiago R. López-Malvar A. Souto C. Barros-Ríos J. (2018). Methods for determining cell wall-bound phenolics in maize stem tissues. J. Agric. Food. Chem. 66, 1279–1284. doi:10.1021/acs.jafc.7b05752 29336154

[B56] Santiago R. Reid L. M. Arnason J. T. Zhu X. Martinez N. Malvar R. A. (2007). Phenolics in maize genotypes differing in susceptibility to Gibberella stalk rot (Fusarium graminearum Schwabe). J. Agric. Food. Chem. 55, 5186–5193. doi:10.1021/jf070641e 17547419

[B57] Schulze-Lefert P. (2004). Knocking on the heaven’s wall: pathogenesis of and resistance to biotrophic fungi at the cell wall. Curr. Opin. Plant Biol. 7, 377–383. doi:10.1016/j.pbi.2004.05.004 15231259

[B58] Segal L. Creely J. J. Martin A. E. Conrad C. M. (1959). An empirical method for estimating the degree of crystallinity of native cellulose using the X-Ray diffractometer. Text. Res. J. 29, 786–794. doi:10.1177/004051755902901003

[B60] Su A. Xiao S. Li Z. Duan S. Wang S. Zhang H. . (2025). Multi-omics analysis elucidates phased defense and resource allocation trade-offs in Fusarium resistance of maize. Plant Stress 17, 100977. doi:10.1016/j.stress.2025.100977 38826717

[B59] Sumarah M. W. (2022). The deoxynivalenol challenge. J. Agric. Food. Chem. 70, 9619–9624. doi:10.1021/acs.jafc.2c03690 35912482

[B61] Swaminathan S. Lionetti V. Zabotina O. A. (2022). Plant cell wall integrity perturbations and priming for defense. Plants 11(24), 3539. doi:10.3390/plants11243539 36559656 PMC9781063

[B62] Tabuchi A. Li L.-C. Cosgrove D. J. (2011). Matrix solubilization and cell wall weakening by β-expansin (group-1 allergen) from maize pollen. Plant J. 68, 546–559. doi:10.1111/j.1365-313X.2011.04705.x 21749508

[B63] Tomassini A. Sella L. Raiola A. D’Ovidio R. Favaron F. (2009). Characterization and expression of Fusarium graminearum endo-polygalacturonases *in vitro* and during wheat infection. Plant Pathol. 58, 556–564. doi:10.1111/j.1365-3059.2008.02019.x 40046247

[B64] Trail F. (2009). For blighted waves of grain: Fusarium graminearum in the postgenomics era. Plant Physiol. 149, 103–110. doi:10.1104/pp.108.129684 19126701 PMC2613717

[B65] Tryfona T. Bourdon M. Delgado Marques R. Busse-Wicher M. Vilaplana F. Stott K. . (2023). Grass xylan structural variation suggests functional specialization and distinctive interaction with cellulose and lignin. Plant J. 113, 1004–1020. doi:10.1111/tpj.16096 36602010 PMC10952629

[B66] Updegraff D. M. (1969). Semimicro determination of cellulose inbiological materials. Anal. Biochem. 32, 420–424. doi:10.1016/S0003-2697(69)80009-6 5361396

[B67] Vorwerk S. Somerville S. Somerville C. (2004). The role of plant cell wall polysaccharide composition in disease resistance. Trends Plant Sci. 9, 203–209. doi:10.1016/j.tplants.2004.02.005 15063871

[B68] Voxeur A. Höfte H. (2020). Pectin-derived immune elicitors in response to lignin modification in plants. Proc. Natl. Acad. Sci. 117, 4442–4444. doi:10.1073/pnas.2000509117 32029583 PMC7060678

[B69] Wang S. Zhang C. Yang J. Wang X. Wu K. Zhang B. . (2020). Sodium butyrate protects the intestinal barrier by modulating intestinal host defense peptide expression and gut microbiota after a challenge with deoxynivalenol in weaned piglets. J. Agric. Food. Chem. 68, 4515–4527. doi:10.1021/acs.jafc.0c00791 32208605

[B70] Wang T. Chen Y. Tabuchi A. Cosgrove D. J. Hong M. (2016). The target of β-expansin EXPB1 in maize cell walls from binding and solid-state NMR studies. Plant Physiol. 172, 2107–2119. doi:10.1104/pp.16.01311 27729469 PMC5129719

[B71] Wang T. Salazar A. Zabotina O. A. Hong M. (2014). Structure and dynamics of brachypodium primary cell wall polysaccharides from two-dimensional 13C solid-state nuclear magnetic resonance spectroscopy. Biochemistry 53, 2840–2854. doi:10.1021/bi500231b 24720372

[B72] Wolf S. Hématy K. Höfte H. (2012). Growth control and cell wall signaling in plants. Annu. Rev. Plant Biol. 63, 381–407. doi:10.1146/annurev-arplant-042811-105449 22224451

[B73] Zang H. Xie S. Zhu B. Yang X. Gu C. Hu B. . (2019). Mannan oligosaccharides trigger multiple defence responses in rice and tobacco as a novel danger-associated molecular pattern. Mol. Plant Pathol. 20, 1067–1079. doi:10.1111/mpp.12811 31094073 PMC6640537

[B74] Zhang B. Gao Y. Zhang L. Zhou Y. (2021). The plant cell wall: biosynthesis, construction, and functions. J. Integr. Plant Biol. 63, 251–272. doi:10.1111/jipb.13055 33325153

[B76] Zhang Y. He J. Jia L.-J. Yuan T.-L. Zhang D. Guo Y. . (2016). Cellular tracking and gene profiling of Fusarium graminearum during maize stalk rot disease development elucidates its strategies in confronting phosphorus limitation in the host apoplast. PloS Pathog. 12, e1005485. doi:10.1371/journal.ppat.1005485 26974960 PMC4790934

[B75] Zhang K. Wang L. Si H. Guo H. Liu J. Jia J. . (2022). Maize stalk rot caused by Fusarium graminearum alters soil microbial composition and is directly inhibited by Bacillus siamensis isolated from rhizosphere soil. Front. Microbiol. 13. doi:10.3389/fmicb.2022.986401 36338067 PMC9630747

[B77] Zhong R. Cui D. Ye Z.-H. (2019). Secondary cell wall biosynthesis. New Phytol. 221, 1703–1723. doi:10.1111/nph.15537 30312479

[B78] Zhou J.-M. Zhang Y. (2020). Plant immunity: danger perception and signaling. Cell. 181, 978–989. doi:10.1016/j.cell.2020.04.028 32442407

[B79] Zuo N. Bai W.-Z. Wei W.-Q. Yuan T.-L. Zhang D. Wang Y.-Z. . (2022). Fungal CFEM effectors negatively regulate a maize wall-associated kinase by interacting with its alternatively spliced variant to dampen resistance. Cell Rep. 41, 111877. doi:10.1016/j.celrep.2022.111877 36577386

