# Supplementary material for: Arabinoxylan and pectin features differentiate maize inbred lines with contrasting Fusarium stalk rot resistance
Source: Front Plant Sci. 2026 Jun 30;17:1840041. doi: 10.3389/fpls.2026.1840041 (PMC13365119; doi:10.3389/fpls.2026.1840041)
Supplement: Supplementary file 1 [file DataSheet1.pdf]

# SUPPLEMENTARY MATERIAL

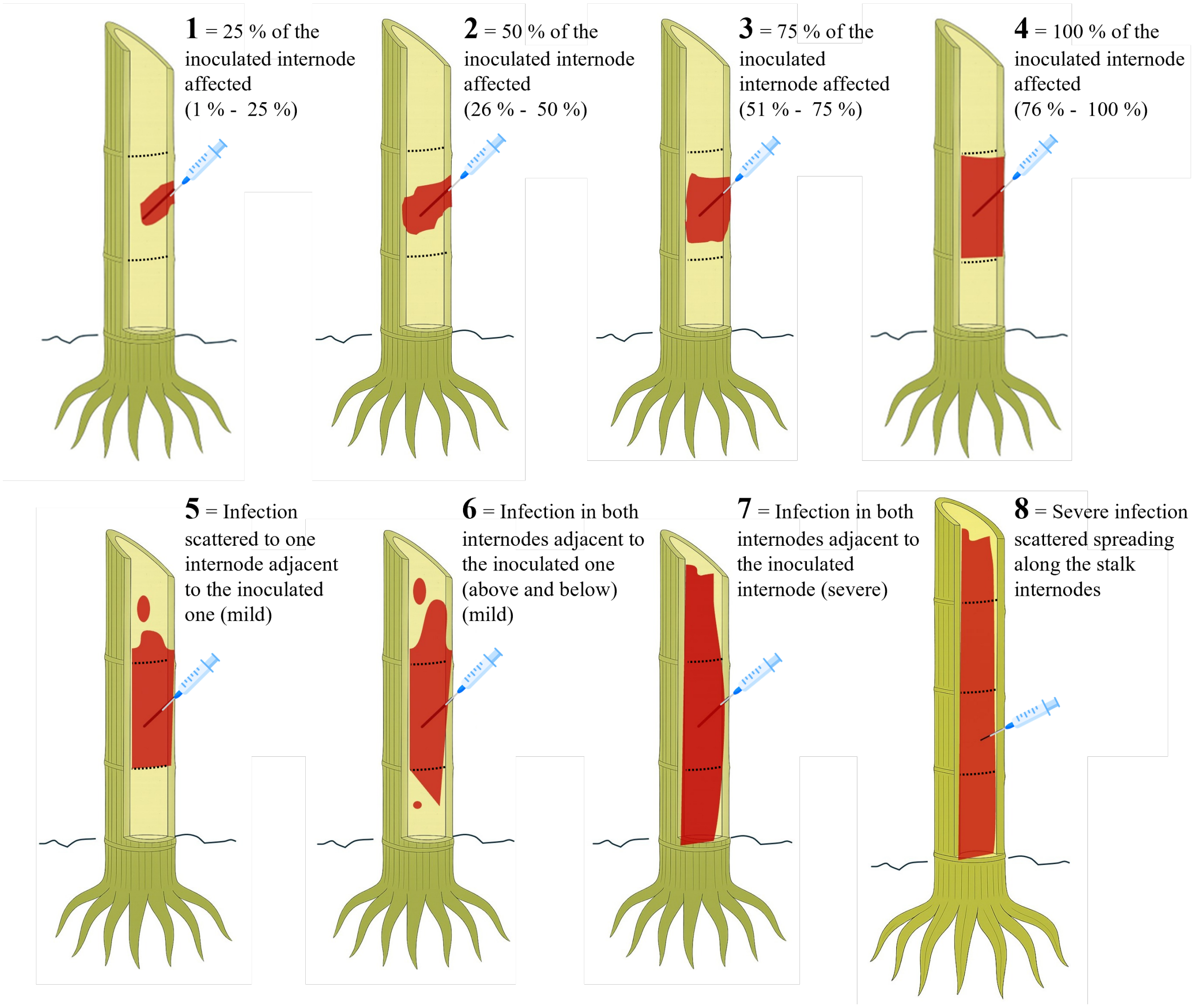


**Figure S1. Damage scale for measuring *Fusarium graminearum* infection through the maize pith,** where: 0 = No spread from the wound area; 1 = 25 % of the inoculated internode affected (1 % - 25 %); 2 = 50 % of the inoculated internode affected (26 % - 50 %); 3 = 75 % of the inoculated internode affected (51 % - 75 %); 4 = 100 % of the inoculated internode affected (76 % - 100 %); 5 = Infection scattered to one internode adjacent to the inoculated one (mild); 6 = Infection in both internodes adjacent to the inoculated one (above and below) (mild); 7 = Infection in both internodes adjacent to the inoculated internode (severe); 8 = Severe infection scattered spreading along the stalk internodes. The inoculated internode (syringe) and the infected area (red) are shown. Scale adapted from Santiago et al., (2007).

A)

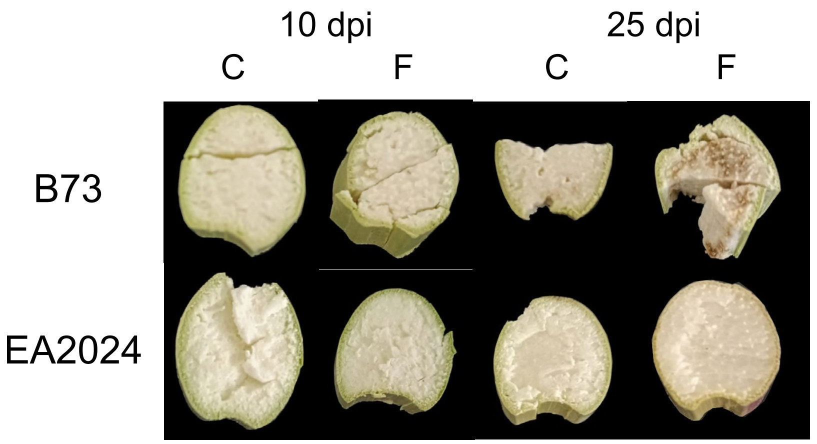


B)

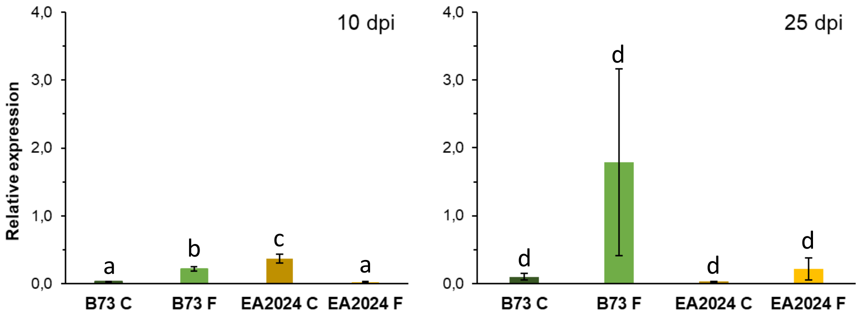


C)

| Primers | Fw | Rev | Tm (ºC) | Cycles (Nº) |
|---|---|---|---|---|
| UBCQ21 | CAGGTGGGGTATTCTTGGTG | ATGTTCGGGTGGAAAACCTT | 60 | 40 |
| 16N | CGGCAGCATACTATGAGGGT | ACTCCTGGAAGCGGAAGGTA | 55 | 40 |

D)

```
>Fusarium graminearum PH-1 chromosome 1
Sequence ID: NC_026474.1 Length: 11697295 bp Range 1: 4718001 to 4718284 bp
Score:525 bits(284) Expect:6e-149 Identities:284/284(100%)  Gaps:0/284(0%)
Strand: Plus/Plus

                  TTCTTTGACATCTGTTCAACCCAGTAAAGCTGTAATAATGTAATTATCAATGACACCTTT
                  ||||||||||||||||||||||||||||||||||||||||||||||||||||||||||||
Sbjct  4718001    TTCTTTGACATCTGTTCAACCCAGTAAAGCTGTAATAATGTAATTATCAATGACACCTTT  4718060

                  GTGTATTCTATCGTTCATCGGCAGCATACTATGAGGGTCTGTTGCATTATCCTTTGTACT
                  ||||||||||||||||||||||||||||||||||||||||||||||||||||||||||||
Sbjct  4718061    GTGTATTCTATCGTTCATCGGCAGCATACTATGAGGGTCTGTTGCATTATCCTTTGTACT  4718120

                  TGAAAAATCTCCTTTATAACTTGTCATATGATACCTTCCGCTTCCAGGAGTTGAAAGCAC
                  ||||||||||||||||||||||||||||||||||||||||||||||||||||||||||||
Sbjct  4718121    TGAAAAATCTCCTTTATAACTTGTCATATGATACCTTCCGCTTCCAGGAGTTGAAAGCAC  4718180

                  AGTCGAACATCATACCTATATACCATTGcattacctacctaggtaggtatccGACATGGC
                  ||||||||||||||||||||||||||||||||||||||||||||||||||||||||||||
Sbjct  4718181    AGTCGAACATCATACCTATATACCATTGCATTACCTACCTAGGTAGGTATCCGACATGGC  4718240

                  AAACTTATAAGTGCAGTTATATGTGCCTGAATCTTGTCATCTGT
                  ||||||||||||||||||||||||||||||||||||||||||||
Sbjct  4718241    AAACTTATAAGTGCAGTTATATGTGCCTGAATCTTGTCATCTGT  4718284
```

**Figure S2. Fungus detection in maize pith tissues by qPCR (A)** Cuts and **(B)** Expression of a conserved fragment of 16N sequence of *Fusarium graminearum* of the second internode of the stem below the main ear of maize inbred lines B73 and EA2024 under control (C) and infection (F) treatments after 10 and 25 dpi. **(C)** Forward (Fw) and Reverse (Rev) primers and conditions (Tm: Melting temperature) used in the qPCR experiment. Relative expression with respect to UBQ21 as housekeeping gene is plotted. Data represent X ± SD (n=3). Different letters indicate significant differences between the samples ($p \leq 0.05$) according to one-way ANOVA followed by Scheffe's post hoc test at 10 dpi, and Kruskal Wallis test at 25 dpi. **(D)** Alignment of the 16N fragment sequence with the *Fusarium graminearum* genome. The yellow sections indicate where the forward and reverse primers align, respectively. Alignment performed using Blastn (megablast) on the NCBI**.**

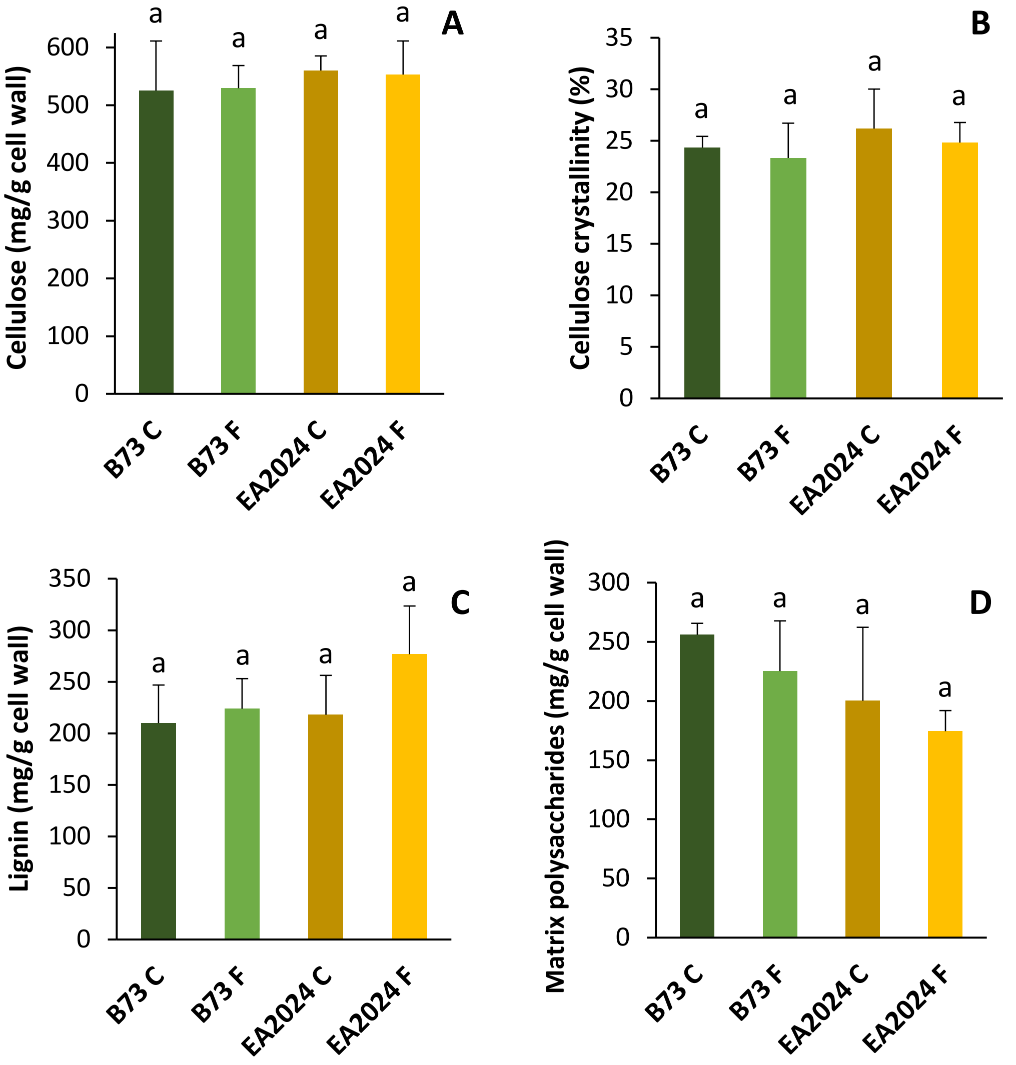


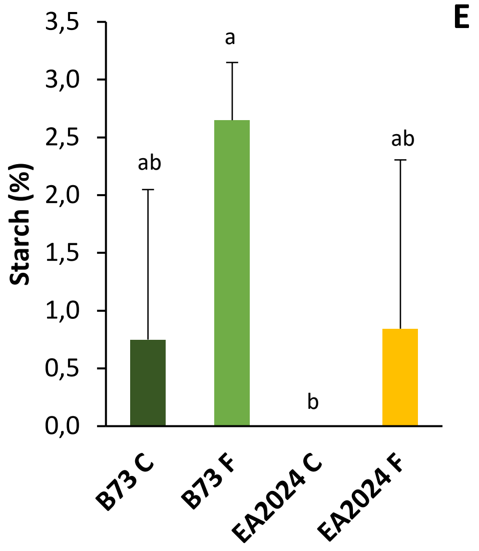


**Figure S3. Major component analysis of pith cell walls from B73 and EA2024 maize inbreds after *Fusarium graminearum* infection. (A)** Cellulose **(B)** cellulose crystallinity, **(C)** Klason lignin, **(D)** matrix polysaccharides and (E) starch contents of pith cell walls from B73 and EA2024 maize inbreds grown in control -C- and *F. graminearum* -F- inoculation conditions. Data represent $\overline{X}$ ± SD (n=3). In each figure, different letters indicate significant differences between the samples ($p \leq 0.05$) according to one-way ANOVA followed by *post hoc* Tukey test.

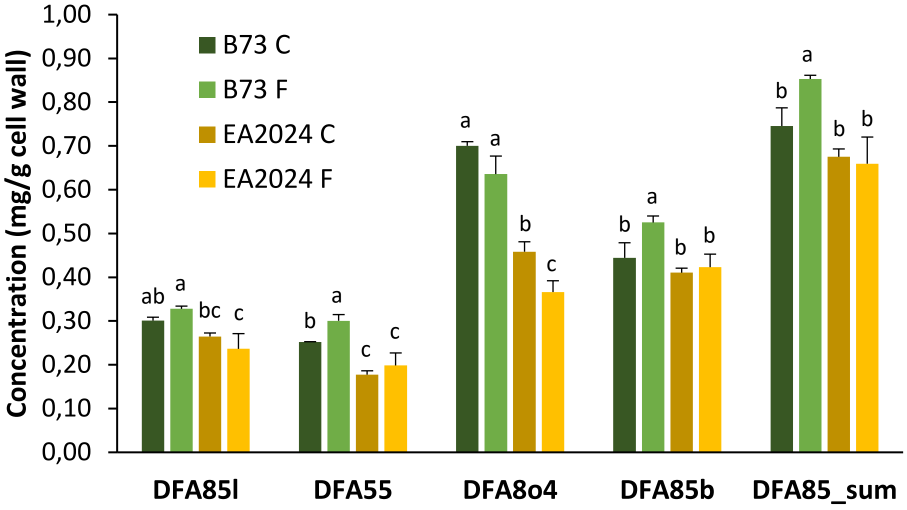


**Figure S4. Content of diferulates (DFAs) of pith cell walls from B73 and EA2024 maize inbreds** grown in control -C- and *F. graminearum* -F- inoculation conditions: DFA85l (8,5′-linear-DFA), DFA55 (5,5′-DFA), DFA8o4 (8–O–4′-DFA), DFA85b (8,5′-benzofuran-DFA) and the total amount of DFA85 (DFA85_sum). Data represent $\overline{X}$ ± SD (n=3). Different letters indicate significant differences between the samples($p \leq 0.05$) according to one-way ANOVA followed by *post hoc* Tukey test.

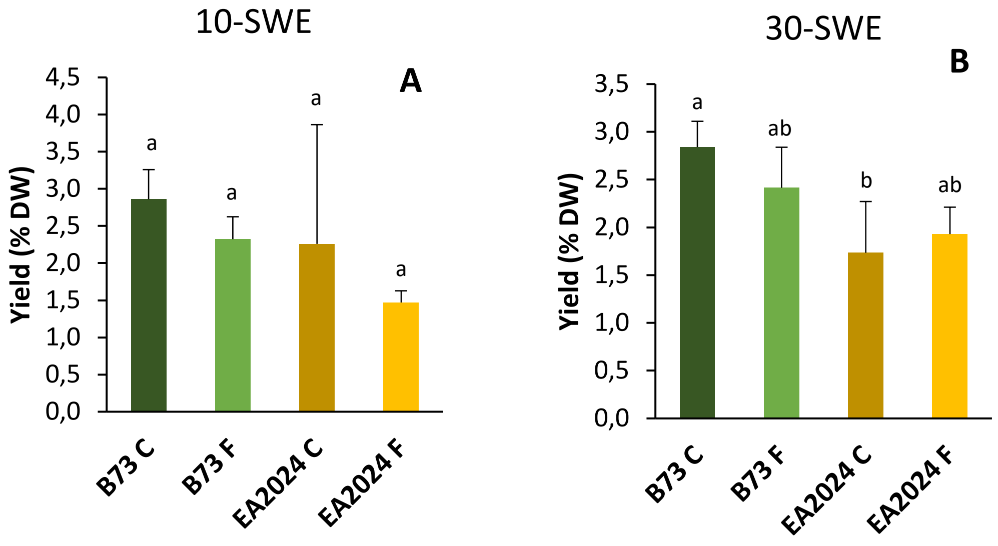


**Figure S5. Gravimetric yields of 10-SWE (A) and 30-SWE (B) fractions of pith cell walls from B73 and EA2024 maize inbreds** grown in control -C- and *F. graminearum* -F- inoculation conditions. DW means dry weight. Data represent $\overline{X}$ ± SD (n=3). In each figure, different letters indicate significant differences between the samples ($p \leq 0.05$) according to one-way ANOVA followed by *post hoc* Tukey test.

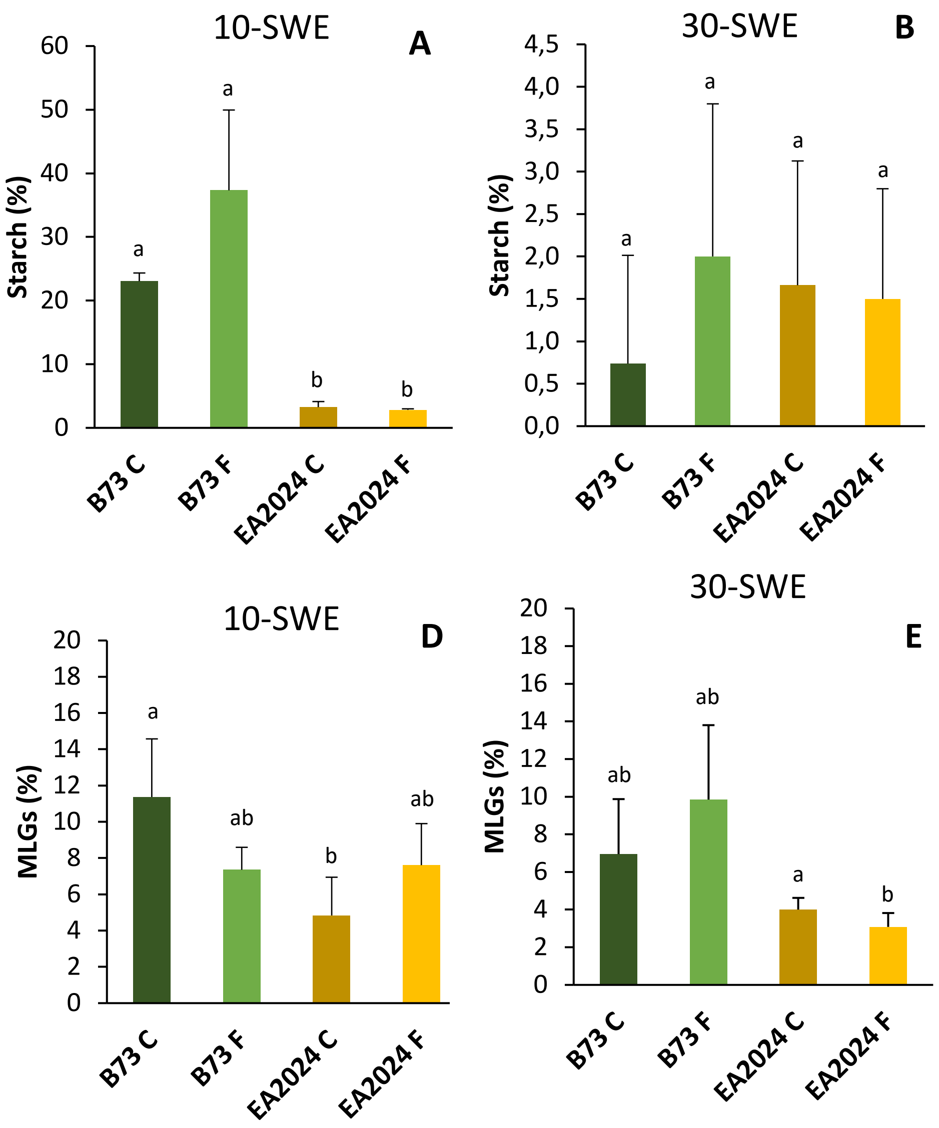


**Figure S6. Starch (A, B) and mixed-linked β-glucans (D, E) percentage of 10-SWE (A,D) and 30-SWE (B, E) fractions of pith cell walls from B73 and EA2024 maize inbreds** grown in control -C- and *F. graminearum* -F- inoculation conditions. Data represent $\overline{X}$ ± SD (n=3). In each figure, different letters indicate significant differences between the samples ($p \leq 0.05$) according to one-way ANOVA followed by *post hoc* Tukey test.
